# Pathogenicity of de novo *CACNA1D* Ca^2+^ channel variants predicted from sequence co-variation

**DOI:** 10.1038/s41431-024-01594-y

**Published:** 2024-03-29

**Authors:** Xuechen Tang, Nadine J. Ortner, Yuliia V. Nikonishyna, Monica L. Fernández-Quintero, Janik Kokot, Jörg Striessnig, Klaus R. Liedl

**Affiliations:** 1 Department of General, Inorganic and Theoretical Chemistry, Center for Molecular Biosciences Innsbruck, University of Innsbruck, A-6020 Innsbruck, Austria; 2 Department of Pharmacology and Toxicology, Center for Molecular Biosciences Innsbruck, University of Innsbruck, A-6020 Innsbruck, Austria

**Keywords:** Genetics, Computational biology and bioinformatics, Diseases

## Abstract

Voltage-gated L-type Cav1.3 Ca^2+^ channels support numerous physiological functions including neuronal excitability, sinoatrial node pacemaking, hearing, and hormone secretion. De novo missense mutations in the gene of their pore-forming α1-subunit (*CACNA1D*) induce severe gating defects which lead to autism spectrum disorder and a more severe neurological disorder with and without endocrine symptoms. The number of *CACNA1D* variants reported is constantly rising, but their pathogenic potential often remains unclear, which complicates clinical decision-making. Since functional tests are time-consuming and not always available, bioinformatic tools further improving pathogenicity potential prediction of novel variants are needed. Here we employed evolutionary analysis considering sequences of the Cav1.3 α1-subunit throughout the animal kingdom to predict the pathogenicity of human disease-associated *CACNA1D* missense variants. Co-variation analyses of evolutionary information revealed residue–residue couplings and allowed to generate a score, which correctly predicted previously identified pathogenic variants, supported pathogenicity in variants previously classified as likely pathogenic and even led to the re-classification or re-examination of 18 out of 80 variants previously assessed with clinical and electrophysiological data. Based on the prediction score, we electrophysiologically tested one variant (V584I) and found significant gating changes associated with pathogenic risks. Thus, our co-variation model represents a valuable addition to complement the assessment of the pathogenicity of *CACNA1D* variants completely independent of clinical diagnoses, electrophysiology, structural or biophysical considerations, and solely based on evolutionary analyses.

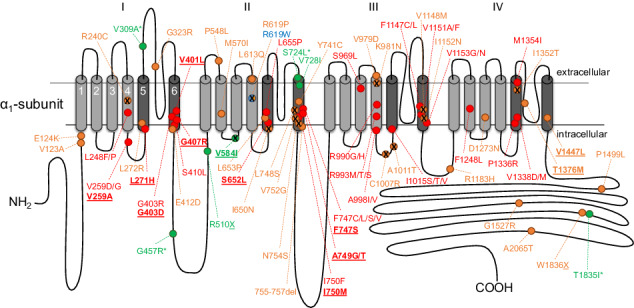

## Introduction

The L-type Cav1.3 isoform of voltage-gated Ca^2+^ channels (VGCCs) controls physiological functions in electrically excitable cells throughout the body, including cochlear inner hair, sinoatrial node, endocrine, and nerve cells [1]. Recently, several reports associated de novo missense variants of *CACNA1D*, the gene encoding the pore-forming α1-subunit of Cav1.3 Ca^2+^ channels, to a wide-spectrum human neurodevelopmental disorder [2–10]. Symptoms include autism spectrum disorder (ASD), but in more severely affected cases also self-aggressiveness, hyperactivity, muscle hypotonia, intellectual impairment, developmental delay, seizures, and in some cases also typical endocrine symptoms at birth (hyperinsulinemic hypoglycemia, hyperaldosteronism). Functional expression of these *CACNA1D* variants revealed characteristic changes of channel gating, predicting enhanced Ca^2+^ influx at subthreshold potentials [11, 12]. Accordingly, enhanced aldosterone production in aldosterone-producing adenomas (APAs) or cell clusters (APCCs) harboring such *CACNA1D* mutations can be explained by enhanced Ca^2+^ signaling driving the activity of aldosterone synthase [13, 14]. The assumption of increased channel activity as disease-causing feature gets further strengthened by the divergent phenotype upon homozygous loss of Cav1.3 function, i.e., deafness and bradycardia (SANDD syndrome, OMIM# 614896) [15, 16]. Since functional consequences of missense variants are more difficult to predict compared to gene-disrupting variants (e.g., nonsense, frameshift, splice-site), we have recently proposed criteria to predict the pathogenic potential of such variants, integrating functional data, information from APAs/APCCs, and criteria proposed by the American College of Medical Genetics and Genomics (ACMG) and the Association for Molecular Pathology guidelines [17].

While this combination of criteria can improve predictions, this approach is highly dependent on available clinical, genetic, and functional information. For pathogenicity prediction of VGCCs, models are mainly based on physicochemical descriptors with structural information [18] or sequence conservation [19, 20]. However, when training pathogenicity models with all types of features included, sequence conservation remains the most dominant one [21, 22]. A short summary of these approaches can be found in SI method section. With the abundant data accumulated in the long history of Ca^2+^ channel evolution [23, 24], the sequence-based approach is particularly interesting for VGCC phenotype predictions. However, while popular methods mostly select sequences by similarity, curated sequences with more functional, and phylogenic concerns are needed to further improve the predictability.

EVcouplings is a sequence-based mutation evaluation tool. On top of the classical single site conservation measurement, it also considers residue–residue couplings, which allow to calculate effects of mutations in the specific context of the target sequence [25]. The method reduces overfitting with the maximum entropy conditions for calculating coupling strengths. Applications of these techniques [26], which detect couplings among residues are summarized in the SI method section. With ever-growing sequence data [27], even better resolution and more coupling information can be expected in the future.

In our study, we apply EVcouplings to well-curated subsets of functionally relevant Ca^2+^ channel α1-subunit sequences throughout the animal kingdom, to evaluate pathogenicity of human disease-associated *CACNA1D* missense variants [11, 12]. We show that this algorithm reliably predicts known pathogenic variants for *CACNA1D*-associated neurodevelopmental disorders and treatment-resistant hypertension, and also improves pathogenicity classification of variants of yet unclear significance.

## Materials and methods

### Sequence collection

**Fig. 1:**
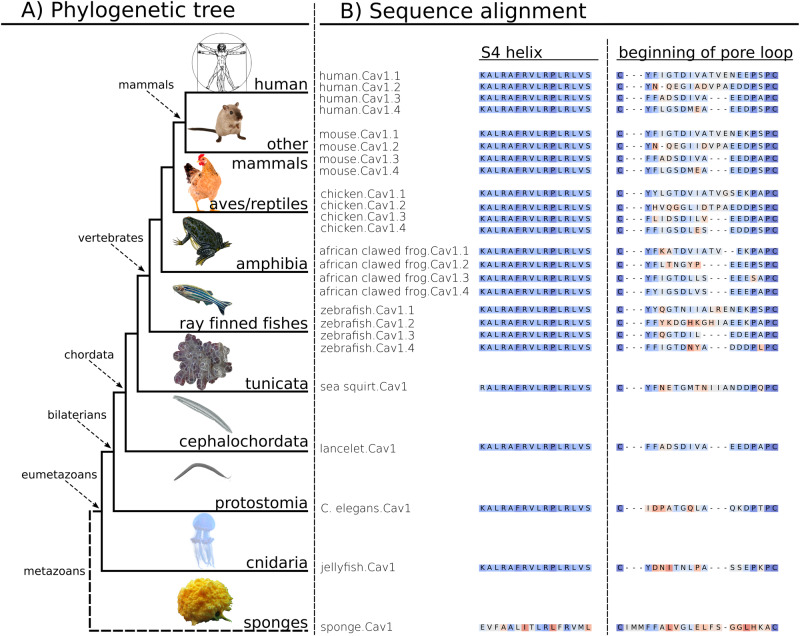
CACNA1D sequence conservation along a simplified phylogenetic tree of animals (Metazoa). **A** Representation of Metazoa phylogeny. Classes and representative branches included in our sequence alignment are indicated with arrows and solid lines. Sponges, which are located outside the “true animal” or Eumetazoa class, diversified much earlier in evolution and are excluded from our alignment (branch in dash line). **B** Examples of sequence alignment of representative species: functionally conserved S4 helices of domain I and the beginning of the variable pore loop between the first two conserved cysteines. Sequences are colored by similarity, identical amino acids and very similar ones are colored in blue, dissimilar ones in red.

Figure 1 shows the *CACNA1D* sequence conservation along a simplified phylogenetic tree of animals (Metazoa). We used the canonical Uniprot amino acid sequence (accession code: Q01668) of *CACNA1D* transcripts to build epistatic and independent models with the EVcouplings tool (v0.1.2) [25]. We started with a sequence search in the Uniref90 database [28] (as it provides a good balance between diversity and reduction of fragment sequences) to collect Ca^2+^ channel sequence ensembles at eight diversification points. This mainly included isoforms in the Eumetazoa (SI Fig. 2) and in vertebrates (SI Fig. 3) of the pore-forming α1-subunits at different levels of complexity: (i) of the whole VGCC family [29] (“model 1”; Cav1–Cav3); (ii) of the high voltage-activated (HVA) Ca^2+^-channel family only (“model 2”; Cav1 and Cav2); (iii) of the L-type subfamily (Cav1) only (“model 3”), and (iv) of the L-type Cav1.3 isoforms only (“model 4”). These ensembles were collected by curating sequences found at various evolutionary distances (measured by bitscore at 0.3, 0.5, 0.7, and 0.8) to study the effect of mutations on the reference sequence. The correct alignment of domains and curation of the sequences to conserve only L-type sequence from the eumetazoan family yields a subset of properly aligned protein sequences with similar functionalities. A mere similarity search would include similar sequences, however, with different functions, e.g., Cav2x sequences in the L-type ensemble. Model 4 with Cav1.3 Eumetazoa sequences also includes invertebrate sequences annotated to be similar to the Cav1.3 subtype. Iterative profile hidden Markov model homology search applying the tool jackhmmer (HMMER 3.3.2 by Eddy et al.) [30], implemented in EVcouplings [25] (https://evcouplings.org), was used to promote the correct alignment of conserved sites. To ensure best coverage of all transmembrane domains, hence eliminating a mismatch of domains in the multiple sequence alignment of the ensemble, we increased the sequence coverage threshold to 70%. Finally, we mapped animal species included in our model to the evolutionary tree derived from the NCBI taxonomy [31, 32] to enable curation by phylogenetics.

Model 3 proved to be the most predictive for pathogenicity and consequently was used for further analyses (for details cf. to the “Choice of the sequence ensemble” section in the SI). The composition of sequences for model 3 is illustrated by Fig. 1. The sequence collection of model 3 possesses a large diversity within Eumetazoa, with 6132 sequences in total. Although condensed on mammalian, avian, actinopterygian, and arthropod channels, sequence composition goes as far back as the cnidarians, around the point where the neuronal and digestive systems essential for pathogenicity of mutations in the humans first appeared [23]. Apart from vertebrates, it also covers numerous protostomia and deuterostomia sequences, where L-type channels covered by model 3 are not split into subtypes. Within the frequently sequenced vertebrate subphylum, highly diversified ray-finned fishes (actinopterygian), birds (aves), and mammals dominate the plot. Cav1.3 sequences of bony vertebrates carrying high sequence identity are downweighted within the class to enhance sequence variations. Variation of sequence similarity along the phylogenetic tree of Metazoa can also be seen with exemplary highly conserved S4 helices and more variable beginning of the pore loop sequences. We colored these sequences from representative species by similarity with BLOSSUM 62 [33] matrix and sum of pairs score [34] for visualization (Fig. 1). In terms of Ca^2+^ channel subtypes, only L-type Ca^2+^ channels are included. All four subtypes Cav1.1–1.4 within the Cav1x subfamily are included in vertebrates. For organisms that evolved into other branches before the vertebrate subtype diversification point, we use corresponding copies of the Cav1x subfamily instead.

### Co-variation model building

We built our epistatic model with pseudo-likelihood maximization calculation (PLMC) [35], to quantify site conservation and residue–residue coupling for reproducing the alignment. We applied 100 iterations of PLMC to minimize statistical noise for coupling terms and positional constraints. Independent models based on maximum entropy were derived without considering coupling context using the same alignment. We calculated differences between mutant and wildtype at each site to score the impact of mutations. Finally, we optimized the sequence identity cutoff of downweighted sequences (0.3 for model 1, 0.6 for model 2, 0.7 for model 3, and 0.8 for model 4 to reduce redundancy and to sharpen the difference between frequently mutated and conserved sites) to avoid that the models are dominated by a large number of similar sequences. Although individual animals within Eumetazoa may have Ca^2+^ channels very different from human ones, the profile hidden Markov model employed by the jackhmmer (HMMER 3.3.2, hmmer.org) prioritized similar ones to include at sequence searching level. Meanwhile, noise minimization and preventions for overfitting in co-variation model building steps implemented in the EVcouplings [25] limited influences of individual outliers. Especially for epistatic models with context-dependent measurements enabled by couplings, model results were more specific toward target species, i.e., human Cav1.3 in our case than a simple average over all species. The basics of the co-variation model building are described in the corresponding section of the SI.

### Electrophysiological measurements

Cav1.3 wildtype (C-terminally long splice variant, Genbank accession number EU_363339, WT_L_) or V584I-containing α1-subunits (V584I_L_) were co-expressed with auxiliary rat β3 (Genbank accession number NM_012828) and rabbit α2δ1 (Genbank accession number NM_001082276), transiently transfected into tsA-201 cells using the Ca^2+^-phosphate-precipitation method and measured with whole-cell voltage-clamp 48–72 h post transfection using 15 mM Ca^2+^ as the charge carrier as described previously [7]. Briefly, WT and mutant channels were recorded in parallel on the same day and data were collected from >3 independent transfections (mean ± SEM). The Ca^2+^ current (*I*_Ca_)–voltage (V) relationship was determined by 50 ms long depolarizations to different test potentials starting from a holding potential of −89 mV (voltages corrected for a liquid junction potential of −9 mV). Steady-state inactivation was determined by calculating the ratio between current amplitudes of a control vs. a test pulse (*I*/*I*_control_; both 20 ms to the voltage of maximal activation, *V*_max_) separated by a 5 s conditioning step to various potentials (10 mV increments; 30 s sweep start-to-start interval; HP, −89 mV) and plotting as a function of voltage. Inactivation kinetics of WT_L_ vs. V584I_L_ were measured during a 5-s depolarization from a holding potential of −89 mV to the voltage of maximal activation.

### Mutation collection and annotations

In line with the wide expression and numerous physiological roles of Cav1.3, genetic *CACNA1D* variants are associated with various human pathologies [12] including a neurodevelopmental syndrome with neurological and endocrine abnormalities (summarized in recent reviews [11, 12]). Somatic mutations were reported in adrenal APAs or APCCs and are associated with increased Ca^2+^-driven aldosterone secretion and treatment-resistant hypertension [36]. These activity-enhancing somatic variants can, therefore, help to predict the pathogenicity of germline mutations if these are found in the same positions. This helps to refine pathogenicity predictions as described previously [11].

We previously classified all reported variants as pathogenic (i) if independently reported in more than one APA/APCC or if typical gating changes were functionally confirmed and no entries were found in controls in gnomAD [37]; or (ii) if present in one control but independently found in more than one APA/APCC with typical gating changes functionally confirmed; or (iii) if absent in controls, reported in one APA/APCC and at least one additional variant (pathogenic/likely pathogenic) reported at the same position [11].

We could, therefore, use known pathogenic variants to further validate our bioinformatic prediction tool and then apply it to variants for which functional data or information from APA/APCC mutations are missing or more uncertain.

We benchmarked our score derived from Eumetazoa L-type Ca^2+^ channels against a well annotated dataset of disease-associated *CACNA1D* variants found somatically in adrenal lesions (APAs, APCCs) or in the germline of patients with a neurodevelopmental disease spectrum [11, 12]. Mutations were mapped to the reference sequence EU363339 as reported recently [11, 12]. EU363339 contains exon 8a but not exons 11, 32, 44, the gnomAD reference sequence NM_000720 contains exons 8b, 11, 32, and 44. For clarity, residue numbers according to the genome reference sequence NM_000720 used in the gnomAD database are also provided in Table 1. We obtained missense variants reported in control groups from the gnomAD [37] (v2.1.1, accessed 01.02.2023, transcript ENSG00000157388) which we use to assess the predictability of likely benign variants and to better understand the relationship between co-variation and pathogenicity. ClinVar Miner [38] (accessed 29.11.2022) mutations scored with pathogenicity not “VUS” were also mapped to histograms of the most predictive model, to test our model on likely pathogenic and likely benign mutation simultaneously with unified selection criteria (cf. SI).

**Table 1:**
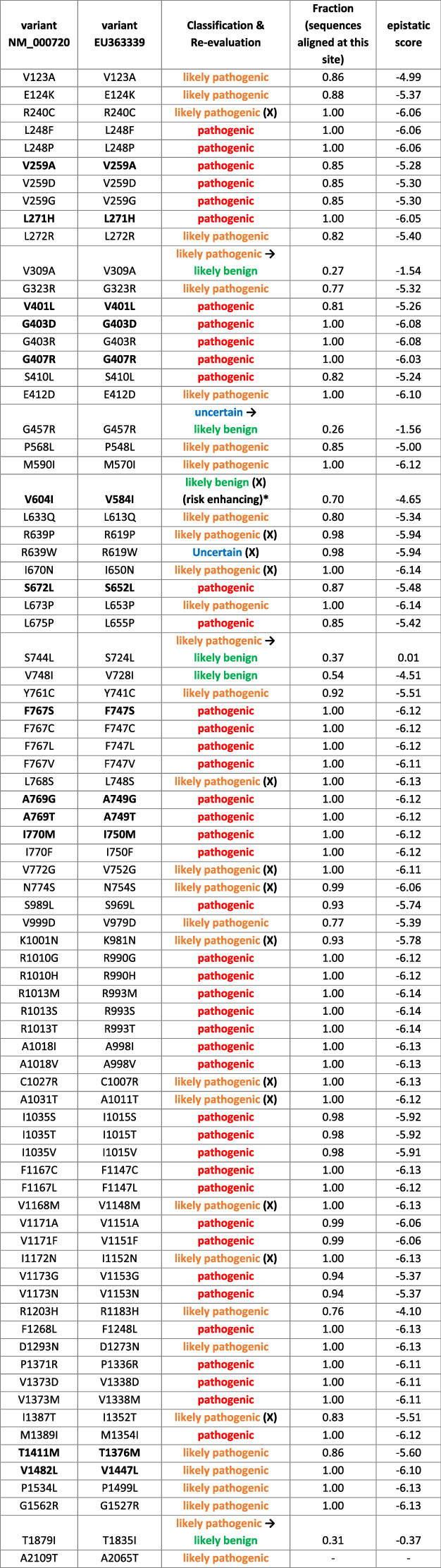
Epistatic score predictions for 80 previously classified CACNA1D variants based on model 3.

The table of variants collected by us previously [2, 3] shows the original pathogenicity annotation as well as statistical details on model predictions. Re-evaluation, i.e., re-classification and re-examination (SI Table 1) are marked with **→** and **(X)**. Reassessment of the literature based on additional criteria triggered by the epistatic score revealed further evidence supporting or not supporting either pathogenicity or non-pathogenicity for some variants as outlined in detail in SI Table 3. De novo CACNA1D variants were either found somatically in adrenal lesions (APAs/APCCs) or in the germline of individuals with a neurodevelopmental disorder (indicated in bold). Residue numbers are given for the genome reference sequence used in the gnomAD database (NM_000720) or in our previous publications (EU363339), which differ due to the incorporation of different exons. The fraction of sequences aligned at the corresponding position scores the certainty of the prediction.

*V584I shows typical pathogenic gating changes (activation at more negative voltages; Fig. 3), however, to a minor extent compared to clearly pathogenic variants, suggesting it to be a risk enhancer rather than a pathogenic variant.

## Results

### Evolution of epistatic heatmap as a function of sequence diversity

**Fig. 2:**
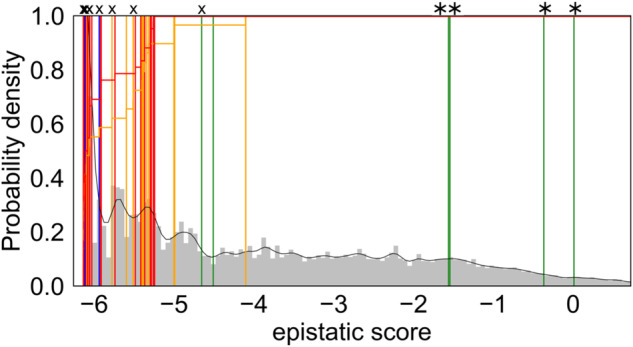
Histograms of the epistatic prediction scores (model 3) of 80 previously published CACNA1D variants with updated pathogenicity classifications (Table 1). The histogram displays the performances of the epistatic model on 80 CACNA1D variants reported somatically in adrenal lesions (APAs/APCCs associated with hyperaldosteronism) or germline in individuals with a neurodevelopmental disorder [11, 12]. Variants were classified into pathogenic (red), likely pathogenic (orange), of uncertain significance (blue) and likely benign (green). These variants are mapped into histograms of the scores of all possible variants calculated for the Cav1.3 α1-subunit sequence as described in “Methods” (aa 68-1888; shown in gray). The overall ratio of pathogenic (red) and likely pathogenic variants (orange) predicted up to a certain score is shown in the histogram (horizontal lines). Four reclassified mutations are indicated with “*” on the top and those re-assessed in terms of the potential risk with “X” (SI Table 3).

In this study, we predicted the pathogenicity of 80 *CACNA1D* variants using EVcouplings on a carefully selected sequence ensemble (Fig. 2). To facilitate the understanding of epistatic scores and heatmaps in the following “Result” section, we provide in Fig. S4 exemplary heatmaps of the epistatic score (combination of site conservation and coupling) for the voltage-sensing domain of repeat III for models 1–4. The plots show clear differences in the respective epistatic scores depending on the model used, highlighting the critical role of sequence selection.

### Predictions of disease-associated *CACNA1D* variants by different models

Figure 2 shows the scoring of 80 previously classified somatic and germline variants [11, 12] by the epistatic model considering evolutionary couplings. All variants are plotted in a histogram according to their calculated score for model 3 (see SI Figs. 1–3 for other models). Scores for model 3 are also summarized in Table 1. The colors of individual lines denote the previously predicted pathogenicity of these variants, which did not take into account any sequence information as described above. Red indicates pathogenic, orange likely pathogenic, green likely benign and blue variants of uncertain significance. Lines on the left side of the histogram are more negatively scored, i.e., representing variants with a large negative impact on fitness and lower probability of occurrence in evolution. Variants on the right side of the histogram are more positively scored and occur more frequently in evolution.

The epistatic model considering evolutionary couplings based on model 3 (Fig. 2) best distinguished the known pathogenic variants. It reliably identified all 42 pathogenic mutations with negative epistatic scores not larger than −5.24 (S410L, Table 1). Notably, also the corresponding independent model (SI Fig. 2) reliably identified all pathogenic mutations with negative epistatic scores not larger than −6.94 (S410L, Table 1). Thus, the choice of the sequence ensemble is more critical for predictivity than the inclusion of the evolutionary couplings for model 3. However, the couplings crucially improve separation of pathogenic mutations from benign ones in models built upon less curated ensembles, i.e., with more sequences of altered functions. This is even more evident when the sequence selection is solely based on the sequence similarity as in models using default parameters of EVcouplings (cf. SI Figs. 1, 2). Although most models (Fig. 2 and SI Fig. 2) find the majority of pathogenic variants in the lower score zone, epistatic model 3 has the least of false negatives (SI Section 2). Moreover, epistatic model 3 also separates well the likely benign mutations from the likely pathogenic ones in the ClinVar Miner dataset on general *CACNA1D* channelopathies (SI Fig. 6).

### Re-assessing pathogenicity classifications in *CACNA1D*

We have previously classified variants using criteria, which were developed along the lines of the ACMG criteria but took into account valuable information on a large number of somatic variants in APA/APCC [11, 12]. We defined variants as likely pathogenic if no functional data were available and they were either reported in only one APA/APCC and were absent in controls, or if reported independently in at least two APAs/APCCs but also one single control. We, therefore, applied the added value of the epistatic score to these likely pathogenic variants. As shown in Table 1 from the 33 likely pathogenic variants for which a score could be calculated (all except A2109T), 26 had an epistatic score within the score range of the pathogenic ones (≤-5.24). This negative score suggested that sequence variation in these positions throughout evolution is associated with decreased fitness and, therefore, increases the likelihood of these variants being pathogenic.

This prompted us to re-examine all likely pathogenic variants for additional evidence of pathogenicity. We defined such evidence (i) if a pathogenic variant had been reported for a conserved amino acid residue in a homologous position in another VGCC α1-subunit or (ii) if data on pathogenic gating changes were reported for site-directed mutations in this position in the literature. Likewise, we searched for evidence that a known pathogenic variant was reported in an APA/APCC in an adrenal with hyperaldosteronism that could explain the phenotype rather than the one rated as “likely pathogenic” in the same adrenal. As shown in SI Table 3, for 12 of the likely pathogenic variants (indicated in bold in the table), we found indeed additional evidence for their pathogenicity, in accordance with their negative score range (−5.51 to −6.14). In contrast, for ten variants we obtained evidence that does not further support their pathogenicity. This is especially true for the four variants with a less negative epistatic score (V309A, −1.54; S724L, 0.01; R1183H, −4.10; T1835I, −0.37). For the other ones, hyperaldosteronism could be explained by the presence of known pathogenic variants in the same adrenal (SI Table 3), although at present this does not rule out their pathogenicity given their negative epistatic score (−5.32 to −6.14).

**Fig. 3:**
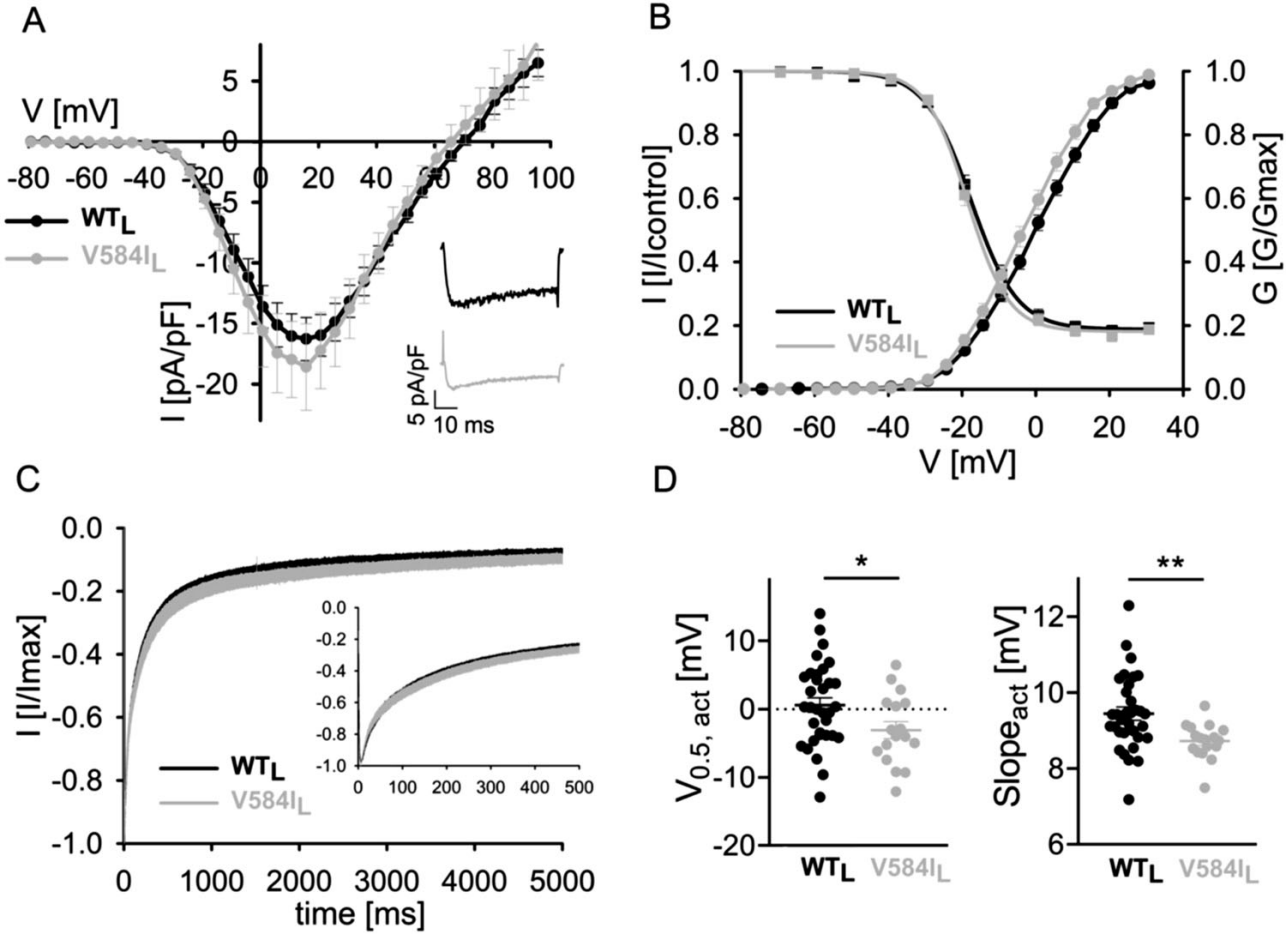
The risk enhancing variant V584I activates at mildly more negative voltages. Whole-cell patch-clamp recordings of the germline CACNA1D variant V584I, which was detected de novo in a male patient with autism from a case-control population [39], were performed as previously described [7]. **A** Ca^2+^ current (*I*_Ca_)–voltage (V) relationship determined by 50 ms long depolarizations to different test potentials starting from a holding potential of −89 mV. Inset: representative *I*_Ca_ traces of WT_L_ and V584I_L_ upon depolarization to the voltage of maximal activation (*V*_max_). **B** Normalized steady-state activation (circles) and inactivation curves (squares) of WT_L_ (black) and V584I_L_ (gray). Statistics are given in SI Table 2. **C** Inactivation kinetics of WT_L_ vs. V584I_L_ during a 5s depolarization from a holding potential of −89 mV to *V*_max_. Inset shows the first 500 ms. Statistics for comparisons of WT_L_ and V584I_L_ at predefined time points are given in SI Table 2. **D** Voltage of half-maximal activation (*V*_0.5,act_) and I–V slope of WT_L_ vs. V584I_L_. Statistics: unpaired Student’s *t*-test.

In addition, for four variants previously classified as likely benign or of uncertain significance, further evidence for pathogenicity was found for negatively scored variants, and evidence for being likely non-pathogenic was found for the more positive scored variant (G457R, −1.56). The score for the likely benign variant V584I was also negative (−4.65, Table 1). It is a germline variant associated with ASD [39] but with eight entries in gnomAD. We, therefore, tested this variant functionally using standard patch-clamp experiments and indeed found a small, but significant gating change compatible with pathogenicity caused by a stronger voltage-dependence of activation (reduced half-maximal voltage and slope factor, Fig. 3, SI Table 2). Since this change was smaller than described for previously characterized pathogenic variants [4, 5, 7–9, 40, 41], it is possible that the less negative score also indicates a lower pathogenic potential (risk enhancer), explaining its lower penetrance.

**Fig. 4:**
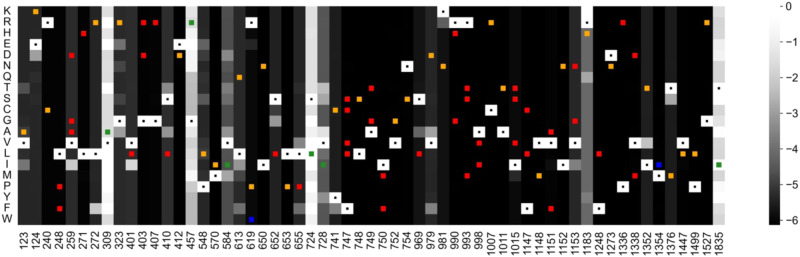
Heatmap of disease-associated CACNA1D mutations with the new classifications. The heatmap of epistatic scores shows consequences of amino acid changes to different residues at positions of reported disease-associated CACNA1D variants [11, 12]. Each column denotes one position while rows represent different amino acid residues at the corresponding position. Resulting boxes are colored according to the epistatic score of the sequence. The darkness reflects the statistical energy for the mutant to appear, i.e., the severity of the predicted impact. As an example, a dark gray box represents a rather unlikely mutant, i.e., predicted with highly negative impact. A lighter box represents a more evolutionarily favorable mutation, i.e., with less negative epistatic score. Boxes corresponding to the reference wildtype sequence are always white and noted with dots. Disease-associated CACNA1D variants are marked with squares with colors corresponding to annotations on pathogenicity. Variants were classified into pathogenic (red), likely pathogenic (orange), of uncertain significance (blue) and likely benign (green), following Table 1.

The heatmap of the epistatic score for mutations collected by us previously [11, 12], based on model 3, is shown in Fig. 4 (for exact scores see Table 1). Like presented in SI Fig. 4, dark gray/black indicates high context-dependent conservation while light gray denotes less negative epistatic impact, i.e., more probability to mutate. The white squares containing a dot indicate the wildtype sequence.

Residue R993, involved in voltage sensing, as also discussed in SI Fig. 4, hardly tolerates any mutation, i.e., all variants (R993S/T/M; Table 1) are predicted to be highly pathogenic shown as dark gray boxes in Fig. 4. Two mutations at G403 with similar pathogenic gating changes are predicted to have the same negative epistatic score. However, some sites show distinct effects for mutations toward different residues, depicted by varying saturation of color along a specific column, e.g., E124 tolerates the charge-conserving mutation to aspartate more than to other amino acids; L613 prefers large bulky and less polar residues like isoleucine, phenylalanine, and to a smaller extent methionine. Therefore, the corresponding mutations are predicted to bear less pathogenic potential than other mutations at the same sites. Finally, some columns have little context-dependent conservation (light colors) and are predicted to tolerate sequence variation without pathogenic potential, such as S724 and T1835. For A2065 no prediction is possible, as sequence heterogeneity allows no proper sequence alignment.

## Discussion

Our recently published collection of 80 disease-associated de novo missense variants of *CACNA1D* [11, 12] allowed us to test our here described models on undoubtedly pathogenic variants. This innovative bioinformatic model purely relies on evolutionary information. As a completely independent approach, it not only reaffirms existing pathogenic classifications but also demonstrates its potential to enhance variant classification, particularly in scenarios with limited conventional criteria.

The pathogenicity predictions of *CACNA1D* variants so far consider several factors, such as the presence at functionally important channel regions, absence in unaffected parents, and healthy controls (gnomAD [37]), recurrence of variants, different variants at the same residue and functional changes. Although a widely used approach, it is highly limited by the available information. For instance, entries in the gnomAD database do not exclude that a certain pathological condition occurred later in the lifespan of an individual, and thus the presence of a certain variant does not automatically exclude a pathological potential. Conversely, APAs/APCCs may harbor other, yet unknown, pathogenic mutations in genes other than *CACNA1D* or other validated risk genes [36]. Adrenal lesions with more than one variant even within *CACNA1D* have been reported, and not all of them may be pathogenic [42]. Thus, the identification of variants in these adrenal lesions does not always imply pathogenicity. Moreover, variants could also be “only” risk-conferring and not disease-causing by themselves, and the genetic background in some patients could also impact the clinical manifestation (e.g., mask or amplify pathogenic effects) [43]. We speculate that this could be the case for the V584I variant for which we found a much smaller but significant negative shift of the activation voltage (−2.5 mV for *V*_0.5,act_ together with a significant decrease in slope factor) than described for previously characterized pathogenic variants [4, 5, 7–9, 40, 41]. Therefore, environment and genetic background may contribute more strongly to the expression of phenotypic alterations thus explaining lower penetrance. It should be noted that while robust gating changes allow to clearly predict pathogenicity, the predictive potential of small gating changes (as for V584I) will require confirmation in more clinically well-characterized patients with confirmed de novo status.

It is important to emphasize that the pathogenicity of variants discussed so far relates to somatic or germline de novo mutations which induce a gain-of-function phenotype. Heterozygous loss-of-functions *CACNA1D* variants are clinically silent [15, 16, 44]. A negative epistatic score could therefore indicate pathogenicity for a loss-of-function variant, which would, however, only manifest clinically in a homozygous or compound heterozygous state. Despite their pathogenicity, such loss-of-function variants could therefore be present at higher allele frequencies in genomes of apparently healthy controls, such as in the gnomAD database [37] (SI Fig. 7).

These ambiguities can be avoided, by analyzing mutations probed by evolution during hundreds of millions of years. For such an analysis it is obviously essential, which sequence ensemble is considered. This is also evident by the fact, that the independent model performs remarkably well, given the right sequence ensemble, even though considering couplings further improves predictions. Both the careful selection of appropriate sequences and the balanced diversity of the sequence ensemble are decisive for the success of predictions.

This becomes apparent by the epistatic scores displayed in SI Fig. 4, that show that conserved sites can be better distinguished from the rest if sequences with larger evolutionary distances are included. However, as the function of included sequences diverges, models including greater diversity in sequences also risk losing specificity of pathogenicity predictions for the target sequence. The model only using the most conserved sequences based on Cav1.3 (model 4) estimates most variants to have very negative epistatic impact (SI Figs. 2, 4). Consequently, effects of individual variants are hardly distinguishable, i.e., scored very similarly due to limited chance of mutation at low evolutionary distance. Models including more diverse sequences, such as the one including HVA channels (model 2) and the one based on L-type channels (model 3), tend to result in larger ranges of epistatic values, balancing good statistics of mutations with the conservation of major biophysical functions (SI Figs. 2, 4).

Similarly, a too low threshold of sequence identity for downweighting similar sequences results in scores dominated by high conservation. A too high threshold increases statistical noise. We observe analogous trends in independent models not considering coupling, but with even less discrimination among neighboring sites. So, a balance between sequence diversity and conserving common functions seems to be essential to the predictiveness of the respective models.

The sequence ensemble of model 3 optimizes pathogenicity prediction by balancing diversity with functional conservation. L-type channels share among its subtypes interactions with β and ɑ2δ subunits as well as Ca^2+^-dependent inactivation [23, 29, 45, 46]. In the meantime, this ensemble also eliminates sequences of the Cav2x protein–protein interaction site, designed for presynaptic vesicle release related interactions [23, 47–50], which are not present in Cav1.3. Largely overlapping functionalities within the L-type channels enhance conservation prediction of related residues, while larger evolutionary distances increase frequency of more neutral mutations. Furthermore, context dependency and hidden Markov model searches in the epistatic effect prediction puts higher weights on the conservation score of the Cav1.3-specific residues via couplings and preferential selection of (functionally) similar sequences. For example, residue S1475 at the C1 helix of the C-terminal domain, only conserved in human Cav1.3 and human Cav1.4 are ranked to be highly conserved in our L-type epistatic model (model 3, score −5.0 for all mutations, but −1.5 for S1475A, with more frequent occurrence in other subtypes). Positions mutating more frequently outside a given subtype are scored more negatively via couplings. This context-dependent scoring helps to correct subtle pathogenicity changes caused by subtype-specific interactions with other proteins. Similarly, species-specific residues can also be recovered via couplings, e.g., likely pathogenic mutations P548L and V123A more frequently appearing in invertebrates are estimated to be more pathogenic, i.e., have more negative relative score and distribute more into the pathogenic peak of the histogram (SI Fig. 2) in the epistatic model. As can be seen in SI Fig. 3, on vertebrate models: vertebrate channels are more similar to the target human Cav1.3, and therefore likely share more common functionalities. However, inclusion of earlier animal sequences drastically improves prediction accuracy and reduces inaccuracy from species-specific functional sites by adding random mutations to sites of less functional relevance or sites specific for highly diversified animal groups.

There are several limitations of the current work and consequently perspectives for future work. First, our model utilizes solely evolutionary information to avoid data leakage leading to train-test contamination. The method is independent of clinical data and the biophysics, including knowledge on structure motives and functional assays providing a complementary perspective. Our model demonstrates high accuracy on pathogenicity assessment when applied alone (SI Section 6). However, appropriate combinations with the orthogonal approaches, available functional annotations and allele frequency in large population sets might enhance accuracy even further. Second, although the coupling term corrects conservation for residues co-evolving with interacting residues for highly variable regions, it still requires good statistics from a minimum percentage of sequence alignment. In regions with frequent gaps, the statistics may not be good enough to evaluate the context-dependent conservation. A column coverage of less than 80% in aligned sequences indicates lower confidence in pathogenicity prediction. In addition, interestingly, our model covers many protein–protein interaction sites that regulate gating currents but are located outside the classical transmembrane domains. This may be due to their constant impact on animal survival. Although trained on evolutionary information only, our model has recognized a list of important functional sites from literature with large number of predicted pathogenic mutations. These sites including a proline-rich domain [51, 52] that binds to A-Kinase anchoring proteins and the PCRD motif in the C-terminal domain, which coordinates cAMP–PK interactions [53, 54]. However, the model may not cover all possible functional sites. Resolution can be lower for functional sites that are only conserved in much closer sequence ensembles to the human *CACNA1D* sequence than the L-type ensemble. Expert knowledge on such sites is needed to form more relevant sequence ensembles which may further improve pathogenicity predictions for these regions.

Taken together, risk prediction of genetic variants strongly depends on available information, which is often limited due to incomplete or undetailed case reports. Thus, our here presented models, which purely rely on evolutionary data, are of great value to complement the assessment of newly identified *CACNA1D* variant, which is crucial for diagnosis and treatment of respective patients.

### Supplementary information

Supplementary Material

Heatmap with Domains

Benchmark with existing methods Table 1

Benchmark with existing methods Table 2

Benchmark with existing methods Table 3

amount of gnomAD variants in different scoring ranges

gnomAD variants scored lower than -4.5 and count >= 1

Predictions for all possible variants

Predictions for VSD III by different models

Rank of APA variants

## Data Availability

Model 3 predictions on all possible *CACNA1D* variants and all Eumetazoa models (models 1–4) predictions on the voltage-sensing domain III are available in the Supporting Information as a ZIP file. Annotation and detailed assessment of the 80 APAs mutations are attached in the manuscript and supporting information. GnomAD (https://gnomad.broadinstitute.org/, v2.1.1, accessed 01.02.2023, transcript ENSG00000157388) with assumed benign variants and ClinVar Miner (https://clinvarminer.genetics.utah.edu/, accessed 29.11.2022, gene *CACNA1D*) data covering both benign and pathogenic variants can be requested online. EVcouplings package is available at https://evcouplings.org or https://github.com/debbiemarkslab/EVcouplings, jackhammer (http://hmmer.org/), and PLMC (https://github.com/debbiemarkslab/plmc) are also accessible online.
