# Supplementary material for: Pathogenicity of de novo CACNA1D Ca2+ channel variants predicted from sequence co-variation
Source: Eur J Hum Genet. 2024 Mar 29;32(9):1065–73. doi: 10.1038/s41431-024-01594-y (PMC11369236; doi:10.1038/s41431-024-01594-y)
Supplement: Supplementary file 1 — Supplementary Material [file 41431_2024_1594_MOESM1_ESM.docx]

# Supplementary Information

# Pathogenicity of de novo *CACNA1D* Ca^2+^ channels variants predicted from sequence co-variation

Xuechen Tang^†^, Nadine J. Ortner^‡^, Yuliia V. Nikonishyna^‡^, Monica L. Fernández-Quintero^†^, Janik Kokot^†^, Jörg Striessnig*^,‡^, Klaus R. Liedl*^,†^

† Department of General, Inorganic and Theoretical Chemistry, Center for Molecular Biosciences Innsbruck, University of Innsbruck, A-6020 Innsbruck, Austria

‡ Division of Pharmacology and Toxicology, Center for Molecular Biosciences Innsbruck, University of Innsbruck, A-6020 Innsbruck, Austria

Email: [Joerg.Striessnig@uibk.ac.at](mailto:Joerg.Striessnig@uibk.ac.at), [Klaus.Liedl@uibk.ac.at](mailto:Klaus.Liedl@uibk.ac.at)

## SI Sections:

1. Basics of co-variation model building
2. Choice of the sequence ensemble
3. Exemplary heatmaps of the epistatic score for the voltage-sensing domain of repeat III and key residues in other domains
4. Benchmark on other datasets
5. Reassessments of 80 previously reported APAs/APCCs mutations
6. Benchmark with existing methods
7. References

## SI Figures:

1. Predictions using EVcouplings default parameters
2. Predictions for different sequence ensembles (model 1-4)
3. Predictions using vertebrate sequences only
4. Heatmap of epistatic score for VSD III
5. Heatmap of epistatic score of other domains
6. Predictions clinVar Miner variants
7. Predictions for gnomAD variants

## SI Tables:

1. gnomAD table
2. experimental measurement table
3. re-examination table

## **Basics of co-variation model building**

EVcouplings calculates both terms by probing site constraints (h) and couplings (J) which best reproduce the multiple sequence alignments. Maximum pseudo-likelihood and maximum entropy approaches help to reduce statistical noise and limit overfitting (with small amounts of sequence per amino acid). Probability P(σ) for occurrence of a sequence (σ) can be recovered from the logarithm of the so-called statistical energy after Boltzmann reweighting (equation 1).

$P(\sigma)=\frac{1}{Z}exp\{E(\sigma)\}$ (1)

The evolutionary statistical energy $E\left( \sigma\right)$ of the sequence σ depends on both site conservation h_i_(σ_i_) of each single site ($\sigma_{i}$) and coexistence of the context by coupling ($J_{ij}(\sigma_{i},\sigma_{j})$) between each pair of residues ($\sigma_{i},\sigma_{j}$) in the sequence (equation 2). Separately, an independent model is derived from the same sequence alignment with site conservation terms calculated under the maximum entropy condition.

$E\left( \sigma\right)=\sum_{i} h_{i}(\sigma_{i})+\sum_{i<j} J_{ij}(\sigma_{i},\sigma_{j})$ (2)

The differences between mutant and wild type are calculated for each possible mutant to score the impact of mutations (equation 3).

$\Delta E(\sigma^{mut},\sigma^{wt})=log\frac{P(\sigma^{mut})}{P(\sigma^{wt})}=E(\sigma^{mut})-E(\sigma^{wt})$ (3)

Bioinformatic tools appear as attractive candidates to further improve pathogenicity predictions^1–6^. Most phenotype prediction tools for proteins in general consider sequence conservation^7–9^, most widely applied predictors such as PolyPhen-2^10^, SIFT^11^, SNAP2^12^ mainly rely on sequence information. Amongst sequence information-derived tools, those detecting the couplings have been widely and very successfully applied to predict effects of mutations^13–19^ and the structure-function relationship of ion channels^20–27^.

## **Choice of the sequence ensemble**

Among all models tested, the epistatic model based on model 3 sequences (SI. Figure 2) seems to best balance diversity and functional conservation. This is evident from the following:

Although most models (Fig. 2 and SI. Fig. 2) score majority of the pathogenic variants rather negatively, the number of false negatives and the separation among variants of different pathogenicity differs among models. For models with very distant sequences, i.e., including sequences of channels with less common functionalities, the conservation of the precise conduction of voltage sensing actions and the current density regulation near the activation gate^28–32^ are compromised on a more regular base (SI.Fig. 2, The clearly pathogenic variant I1015V is ranked less pathogenic in both the model 1 and model 2, and F747L neutral in model 1). On the other hand, for the model 4 (SI.Fig. 2) containing the most conserved sequences, scarce statistics dominates the pathogenicity ranking, originating from random mutations in more distant species to humans. Consequently, well-documented pathogenic variants such as A998I and I1015V are wrongly scored less pathogenic, erroneously implying that they would have similar occurrence as less pathogenic variants. However, comparing with the independent model built upon the same alignment, it can be observed that the coupling improves the scoring by considering the presence of interacting residues in the human target sequence. Consequently, several pathogenic variants are incorrectly classified as less pathogenic and shifted into the more neutral zone.

The epistatic models, which consider the couplings also tend to correct scoring of wrongly classified pathogenic variants towards more negative ranges. On the contrary, independent models (SI. Fig. 2) built upon the same sequence ensemble miss more functionally important sites and show less separation between pathogenic and benign mutations. This effect is particularly evident in more diverse models, e.g., in the HVA model 2 (SI. Fig. 2). The likely pathogenic mutation Y741C and the pathogenic mutation I1015V are predicted to have more negative epistatic impact, i.e., to be more pathogenic in the epistatic model on HVA channels (model 2). For example, “likely pathogenic” mutations P548L and V123A are predicted to be more pathogenic in the epistatic model based on L-type channels (model 3), while scoring similarly to benign mutations in the independent model based on the same sequences.

Since our model is also able to identify couplings between residues, it is interesting to note that variant I1015V may affect gating due to coupling of I1015 in IIIS5 to G1149 located within the activation gate formed by IIIS6. Variant A998V might be an example of loss of conduction. Residue A998 joins the III S4 with the III S4-S5 linker and is in close proximity to the IV S5 in the pore domain. As A998I is already frequently occurring in human Cav2x, the sterically less demanding substitution A998V is predicted by model 2 to be more prone to occur. Finally, model 3 shows the best separation between pathogenic and benign variants. The L-type channel ensemble includes sequences that conserve a large number of common functions from Cav1.3 (auxiliary subunit interaction, CDI etc.) and more completely includes animals lower than vertebrates with different subtype diversifications. These lower animals already show biological systems similar to humans (e.g., neurons and the digestion system) ensuring conservation of residues related to target human Cav1.3 channel regulations. Meanwhile, a large number of diverse sequences originating from different subtypes and organisms allow for a more complete test of interchangeable residues along the channel. Still the statistics is not sufficient for discrimination of effects and couplings at very conserved and functionally relevant sites. Among all models tested, the epistatic model based on ‘eumetazoa’ L-type calcium channel sequences (model 3) seems to best balance diversity and functional conservation. Hence, the following discussion focuses on this model to illustrate the relationship between clinical phenotypes and epistatic prediction.

In summary, a well-adjusted equilibrium between frequent mutations of less functionally relevant sites (promoted by sequence diversity) and constraints on shared functionalities, i.e., the regions executing them, is required for a good correlation of predictions with actual pathogenicity.

Although the model basing on HVA (model 2) performs similarly to the L-type model (model 3) in total outlier count and even shows more distinguishable peaks, additional pathogenic variants are missed by the model which were found in distinct glands from independent/individual studies. These outliers can even have pathogenic variants found at the same sites, classifying them as significant outliers. These missing points mark the appearance of additional conserved/functional sites in Cav1.3 when diversifying to HVA (model 2).

Additionally, residues with predicted pathogenic mutations by the model basing on L-type channels (model 3) cover known functional sites, shown with an example of the whole domain I (SI. Fig. 4). Model 3 efficiently sorts out functionally important residues from the rest, e.g., for the voltage sensor depicted, counter charges reported to modify the electrophysiology and important for gating properties in the Cav1.1 channel^33,34^ are all marked with predicted extremely pathogenic mutations (score < -5.8), while other negatively charged residues in the voltage sensor mostly hold predicted benign mutations.

*
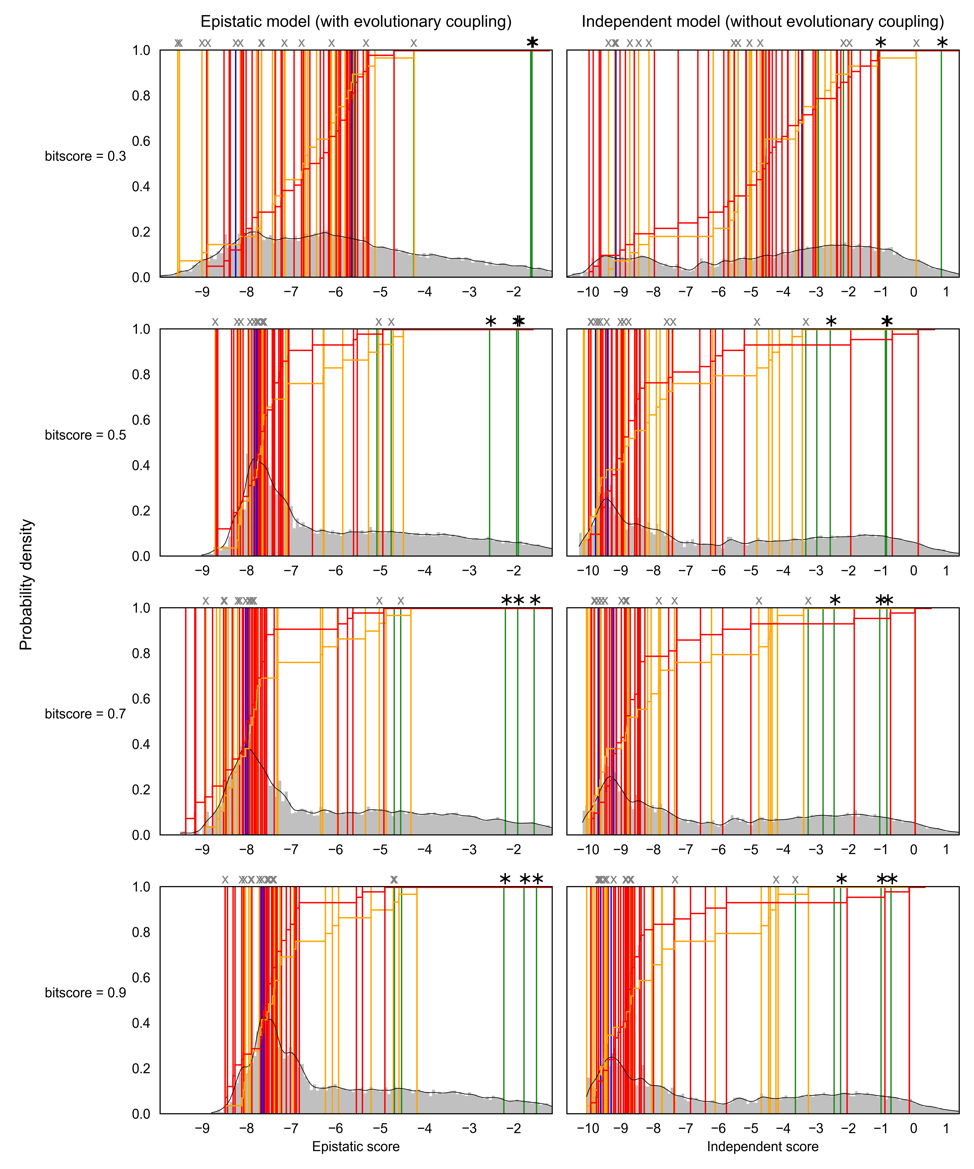
****Supplemental Figure 1: Predictions for CACNA1D variants for which pathogenicity has been classified previously by models using default parameters of EVcouplings and different similarity cutoffs (bitscore)*, basing the sequence ensembles solely on sequence similarity.**

*The histograms display performances of the four epistatic (left) and independent models (right) on 80 CACNA1D variants reported somatically in adrenal lesions (APAs/APCCs associated with hyperaldosteronism) or germline in individuals with a neurodevelopmental disorder*^29,35^*. Models using default parameters of EVcouplings result in ensembles of less functional relevance, which lead to less separation between pathogenic and benign mutations. Variants previously reported in the literature were reclassified with reassessment of the literature (Tab. 1) into pathogenic (red), likely pathogenic (orange), of uncertain significance (blue) and likely benign (green). These variants are mapped into histograms of the scores of all possible variants calculated for the Cav1.3 a1-subunit sequence as described in Methods (aa 68-1888; shown in grey) to correlate the predicted pathogenicity with epistatic scores. The overall ratio of pathogenic (red) and likely pathogenic variants (orange) predicted up to a certain score is shown in each of the histograms (horizontal lines). From top to bottom, as the similarity cutoff for sequence alignment gets closer to the target sequence, the sequence ensemble collected becomes less diverse and more similar to the target sequence. Although at the bitscore=0.7 similarity cutoff, the epistatic model results in reasonable separation between pathogenic and benign mutations, some clearly pathogenic mutations colored in red are scored rather neutral. Meanwhile, scoring and distribution with respect to the background mutation distribution of the two groups are rather similar. The close distribution of pathogenic and benign mutations and more neutral score of clearly pathogenic mutations raise concern on generalizability of this score for variants and their classifications outside the benchmark mutation set.*


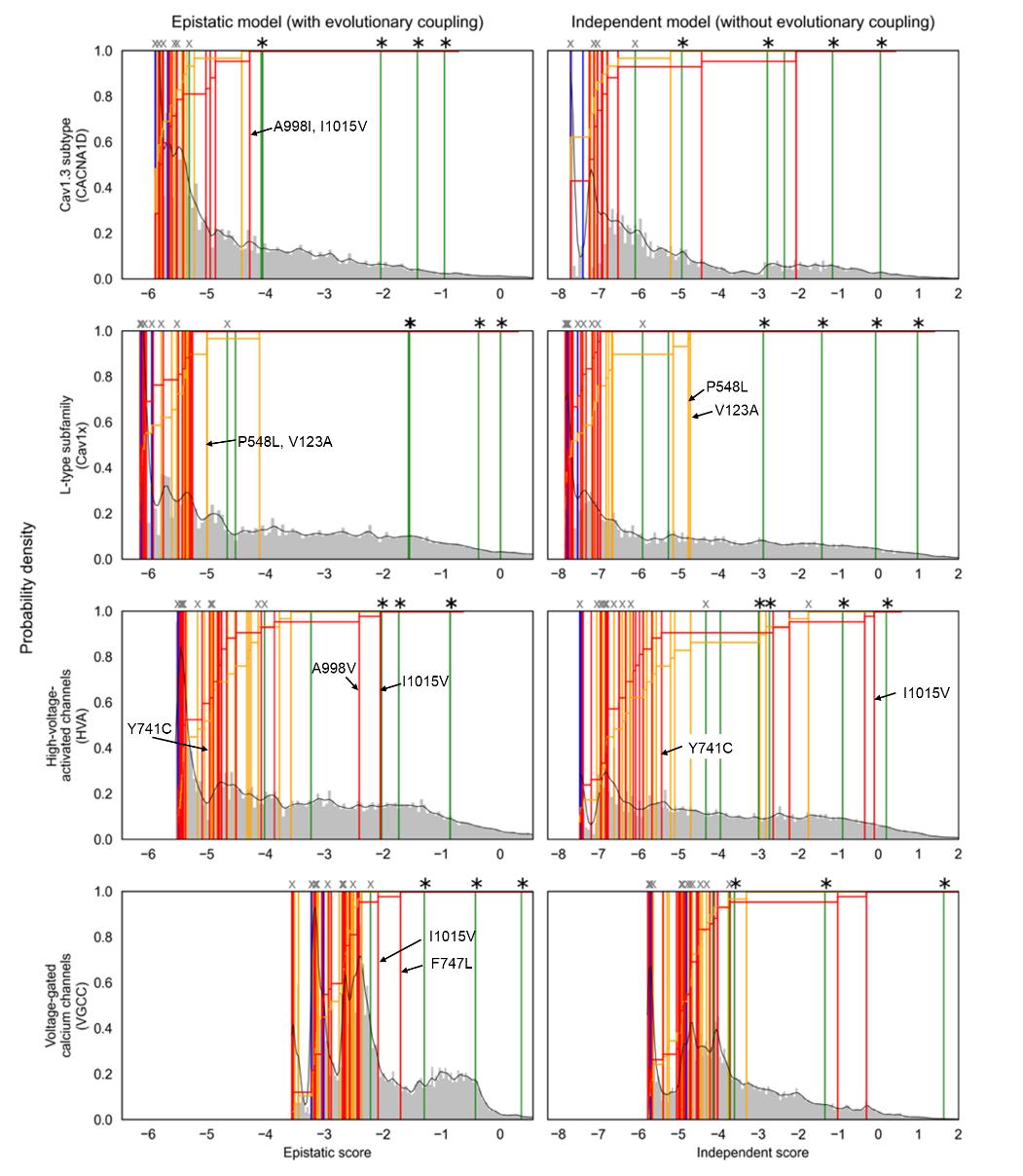


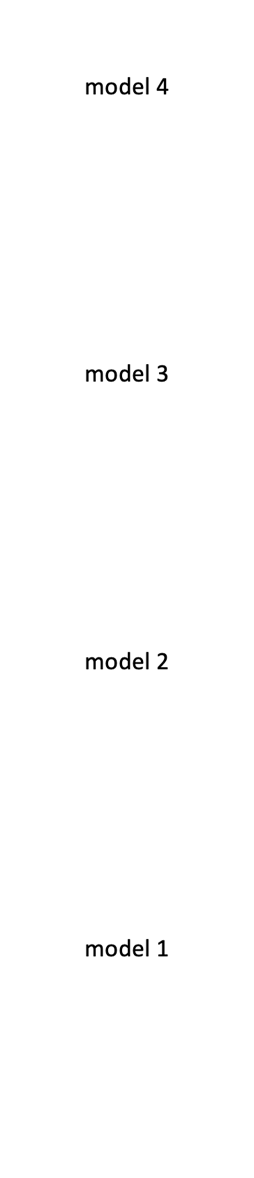
***Supplemental Figure 2: Histograms of the epistatic (left) and independent (right) prediction scores for four different sequence ensembles (model 1-4) mainly basing on eumetazoa sequences with CACNA1D variants for which pathogenicity has been classified previously.***

*The histograms display performances of the four epistatic (left) and independent models (right) on 80 CACNA1D variants reported somatically in adrenal lesions (APAs/APCCs associated with hyperaldosteronism) or germline in individuals with a neurodevelopmental disorder*^29,35^*. The sequence ensembles comprise: the whole voltage-gated Ca^2+^ channel (VGCC) family*^36^ *(“****model 1****”; Cav1-Cav3); the high voltage-activated (HVA) Ca^2+^-channel family only (“****model 2****”; Cav1 and Cav2); the L-type subfamily (Cav1) only (“****model 3****”) and the L-type Cav1.3 isoforms only (“****model 4****”). Variants were previously reported in literature were reclassified with reassessment of the literature (Tab. 1) into pathogenic (red), likely pathogenic (orange), of uncertain significance (blue) and likely benign (green). These variants are mapped into histograms of the scores of all possible variants calculated for the Cav1.3 a1-subunit sequence as described in Methods (aa 68-1888; shown in grey) to correlate the predicted pathogenicity with epistatic scores. The overall ratio of pathogenic (red) and likely pathogenic variants (orange) predicted up to a certain score is shown in each of the histograms (horizontal lines).*

*
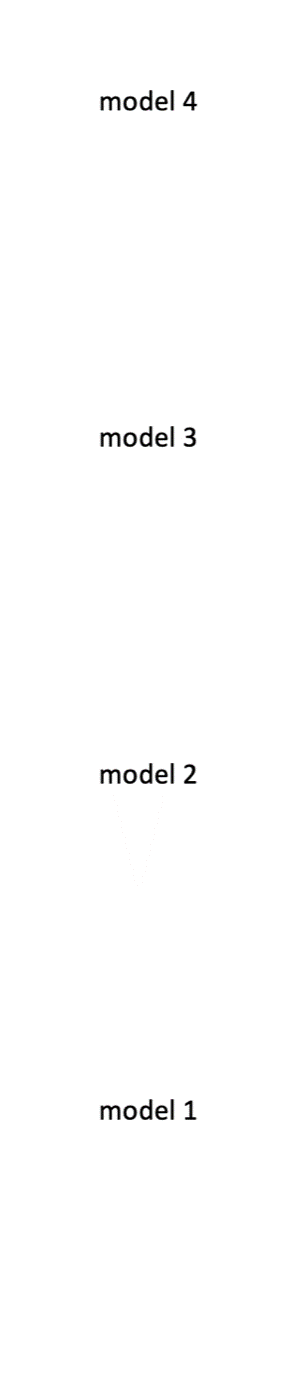
*
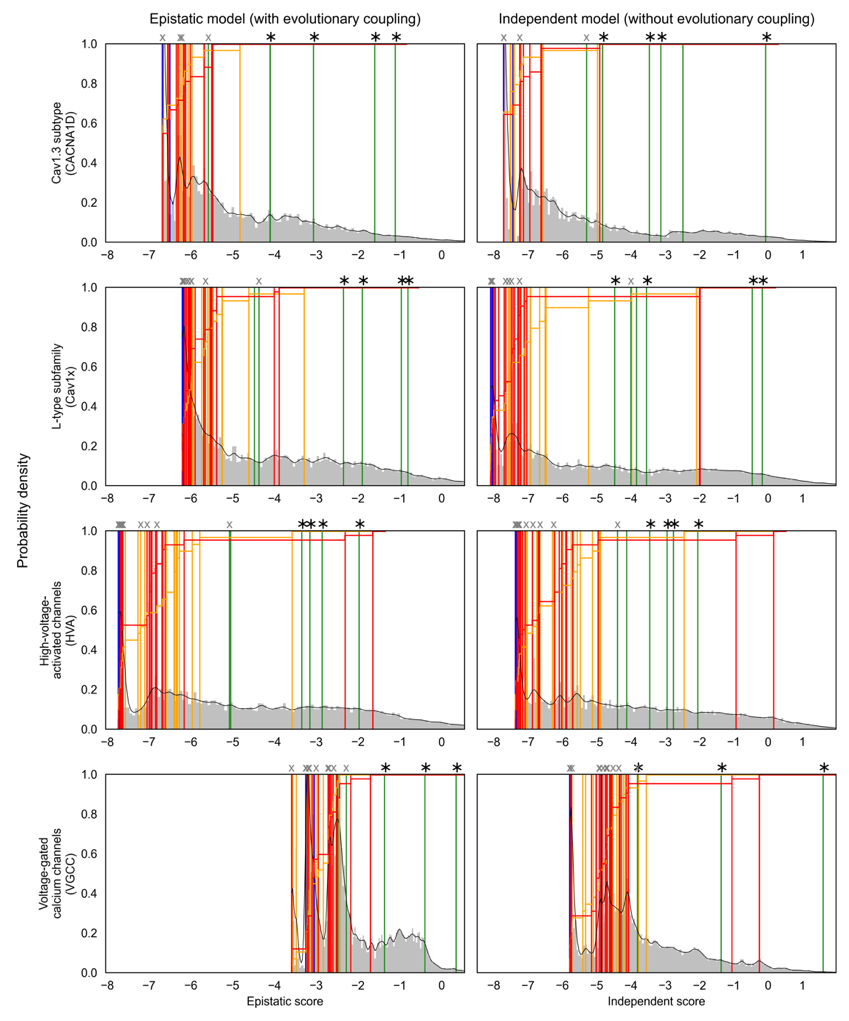
***Supplemental Figure 3: Predictions by different models basing on vertebrate sequences only on CACNA1D variants with the new classifications.***

*The histograms display performances of the four epistatic (left) and independent models (right) on 80 CACNA1D variants reported somatically in adrenal lesions (APAs/APCCs associated with hyperaldosteronism) or germline in individuals with a neurodevelopmental disorder*^29,35^*. Variants were previously reported in literature are reclassified with reassessment of the literature (Tab. 1) into pathogenic (red), likely pathogenic (orange), of uncertain significance (blue) and likely benign (green). These variants are mapped into histograms of the scores of all possible variants calculated for the Cav1.3 a1-subunit sequence as described in Methods (aa 68-1888; shown in grey) to correlate the predicted pathogenicity with epistatic scores. The overall ratio of pathogenic (red) and likely pathogenic variants (orange) predicted up to a certain score is shown in each of the histograms (horizontal lines). Models with vertebrate sequences only are generally less predictive than those with eumetazoa sequences (SI.Fig. 2).*

## **Exemplary heatmaps of the epistatic score for the voltage-sensing domain of repeat III and key residues in other domains**

To facilitate the understanding of epistatic heatmaps, SI Figure 4 contains the heatmaps of the epistatic score (combination of site conservation and coupling) for the voltage-sensing domain of repeat III (VSD III, including transmembrane helices S1-S4) for the models based on the α1-subunits of all voltage-gated Ca^2+^ channels (model 1, VGCCs); the high-voltage-activated Ca^2+^ channel family (model 2, HVA; Cav1 and Cav2); the L-type subfamily and the Cav1.3 channels (model 4). This region was selected to demonstrate differences in the epistatic scoring for several conserved residues required for proper channel function throughout different models.

Voltage-sensing by the channel depends on the outward movement of the S4 helix in each of the four domains upon depolarization. Voltage is sensed by highly conserved positive charges in S4 (K981, R984, R987, R990, R993, R997; SI. Fig. 4), which requires interaction with negative counter-charges in other helices during transmembrane movement, including D923 and E933 in IIIS2^28^. In addition, the voltage-sensor needs to be sealed against permeating ions by conserved phenylalanines in each S2 helix (F930 in domain III) to form a hydrophobic constriction site which prevents cation permeation through the sensor.

As illustrated in SI. Figure 4B, inclusion of more isoforms in the calculation of the epistatic score (from left to right) increases evolutionary distance and sequence diversity and therefore also increases the probability of mutations and of amino acid replacements to be tolerated in a particular position. However, these functionally important residues show a very negative epistatic score in all models (SI. prediction_VSD_III.xlsx excel table) indicating that mutations to any other residue would reduce the fitness of the channel.

K981, tolerates variation to arginine, which conserves the functionally important positive charge with a less negative epistatic score (-4.36; vs. <-5.8 for all other residues in the L-type Ca^2+^ channel model 3, see SI. prediction_VSD_III.xlsx excel table). This is also true for mutation of R997 to lysine (Cav1.3-model 4) or even to glutamine (HVA-model 2) only partially preserving the charge. The epistatic score becomes less negative for R993 as the sequence diversity increases in the HVA and the VGCC-models (model 2 and model 1) but still retains high context-dependent conservation.

No replacements appear to be tolerated in F930 and in the negative countercharges of HVA channels (model 2). Countercharge D923 tolerates (albeit with a negative epistatic score) a mutation to the hydrogen bond forming analogue asparagine only in the more distant HVA- and VGCC-models (model 2 and model 1). However, all other variations are predicted to be disruptive by all models.

The mutation R990H has been verified as pathogenic by functional studies complemented by molecular dynamics simulations and R990G (4x reported as somatic mutation, absent in gnomAD) was considered pathogenic as well (Monteleone et al., Ortner et al. 2020). R990H permits ion flux through the voltage sensor by disrupting the seal around the hydrophobic constrictions site (Monteleone et la. 2017). This is in line with the very high context-dependent conservation of this position in all models tested (SI.Fig. 4B, SI. prediction_VSD_III.xlsx excel table).

M960 is illustrated as an example of a residue with low context-dependent conservation. The co-variation analysis even suggests a positive epistatic score for replacing this methionine with a leucine consistently in all four models. In the Cav1.3-model (model 4, SI. Fig. 4B), mutations to alanine and serine are predicted to be possible, but with a small negative epistatic score. In the more diverse models, other substitutions also appear tolerated but small preferences vary within models.

Regions of higher sequence diversity are especially the loop regions connecting the four helices S1-S4 (SI. Fig. 4). Even positions with low homology such as residues 909-912 in S1-S2 and Q973 in S3-S4 show large difference in epistatic scores, indicating clear preferences towards subsets of residues while avoiding others. This can help to distinguish variants with a higher probability for increasing disease risk from tolerated replacements.

The epistatic scores displayed in Figure 2B clearly demonstrate, that conserved sites can be better distinguished from the rest if sequences with larger evolutionary distances are included. However, as the function of included sequences diverges, models including greater diversity in sequences also risk losing specificity of pathogenicity predictions for the target sequence. The model only using the most conserved sequences based on Cav1.3 (model 4) estimates most variants to have very negative epistatic impact (SI. Fig. 4B, SI. Fig. 2). Consequently, effects of individual variants are hardly distinguishable, i.e., scored very similarly due to limited chance of mutation at low evolutionary distance. Models including more diverse sequences, such as the one including high voltage activated channels (model 2) and the one based on L-type channels (model 3), tend to result in larger ranges of epistatic values, balancing good statistics of mutations with the conservation of major biophysical functions (SI. Fig. 4B). Functionally important residues, such as the S4 charges (K981, R984, R987, R990, R993 and R997) responsible for upward movements upon depolarization and F930 which avoids leakage and controls kinetics in the hydrophobic constriction site, now show clearly different scores compared to less significant residues, e.g., loop residues. (The Phe in the hydrophobic constriction site and positively charged residues on the S4 helices are scored similarly in other domains. Heatmaps of predicted scores from model 1-4 are shown in SI. Fig. 5) As the diversity of sequences goes up even further with the most diverse model basing even on the entire VGCC family (model 1), only very few sites stand out from the statistical epistatic background noise (SI. Fig. 4B). Similarly, a too low threshold of sequence identity for down-weighting similar sequences results in scores dominated by high conservation. A too high threshold increases statistical noise. We observe analogous trends in independent models not considering coupling, but with even less discrimination among neighboring sites. So, balance between sequence diversity and common function conservation seems to be essential to the predictability of models.

The heatmaps derived from the evolution data also show additional information, compared to the table of residue conservation with solely human sequences of different subtypes. For example, the counter charge D923 proven to regulate the gating behaviors^1,37^ and the R915 absent of reported pathogenic variations or functional studies show the same conservation level among human subtypes, i.e., both are conserved among the HVA channels and mutated in T-type channels^38^. However, the R915 mutation is generally predicted with a less negative score and tolerates more mutations than D923 in our heatmaps.

*
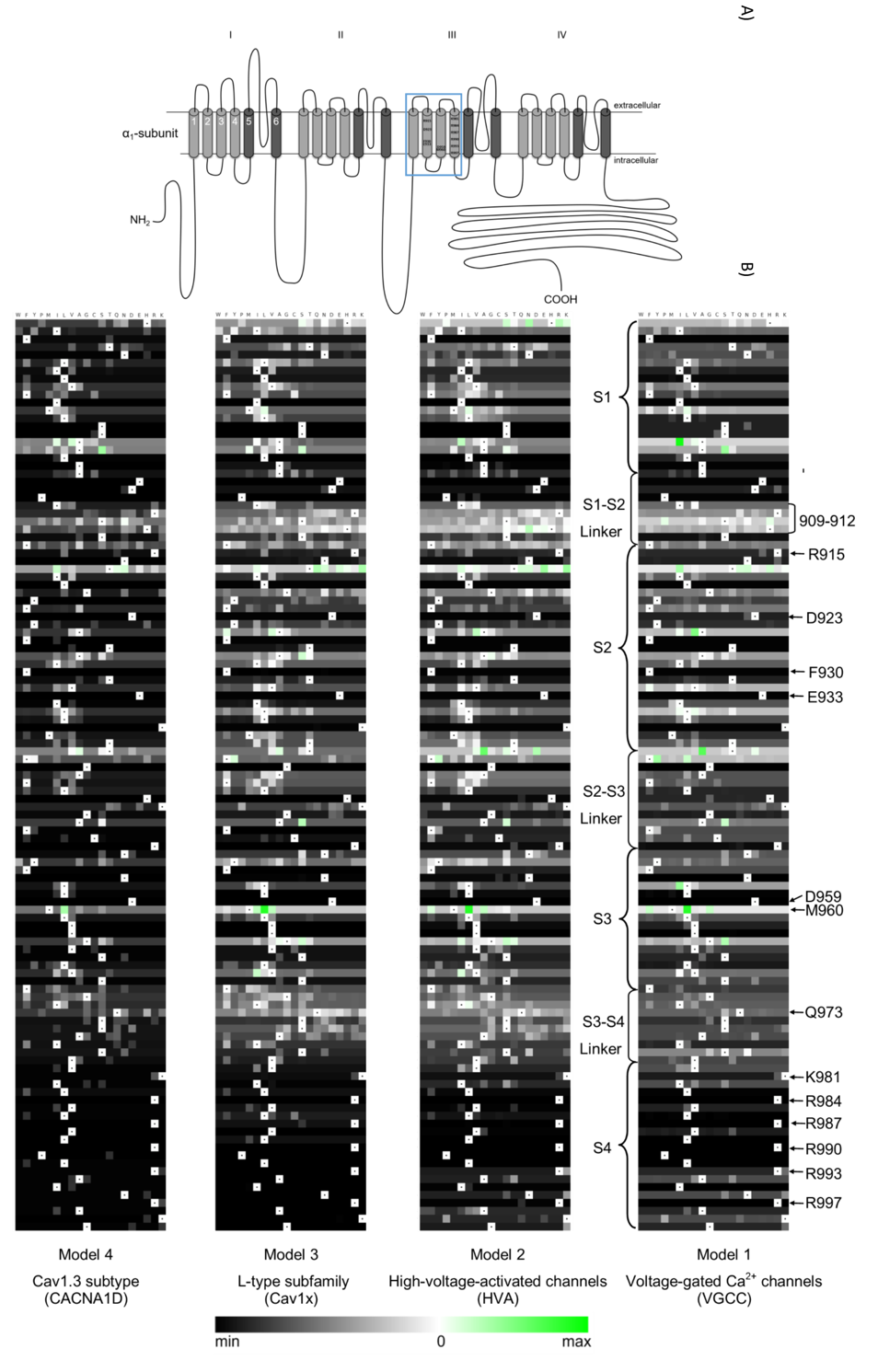
****Supplemental Figure 4: Heatmap of models at different diversification points (VSD III).***

1. *Transmembrane topology of the Cav1.3 α1 subunit, with its four homologous domains (I-IV) each consisting of six transmembrane segments (S1-S6). S1-S4 form the voltage-sensor modules. All four S5, S6 helices and their S5-S6 linkers form a single Ca^2+^-selective pore. The voltage-sensing domain of repeat III (VSD III) shown in the heatmap is highlighted (blue box). The residues analyzed in detail are marked at their approximate positions on corresponding helices (gray bars). B) The heatmaps show epistatic scores of all possible mutations in the VSD III, predicted by models based on different sequence ensembles as indicated at the bottom. Each row represents one amino acid position, with 20 columns indicating the probability of another residue (given in single amino acid letter code on top) replacing the wild-type residue (white dotted square). The darker the box, the more negative the predicted epistatic impact (i.e., high context dependent conservation) with higher probability that the mutant sequence is putatively deleterious. With inclusion of more isoforms the darkest gray corresponds to the minimum of the epistatic score and the darkest green the maximum of the epistatic score within each model respectively. Green boxes indicate a positive epistatic score, i.e., low context dependent conservation. The diversity of sequence ensembles for epistatic predictions increases from top to bottom model 1, all VGCCs (Cav1-3); model 2, all high-voltage-activated Ca^2+^ channel a1-subunits (Cav1 and Cav2); model 3, L-type Ca^2+^ channel a1-subunits (Cav1); model 4, Cav1.3 a1-subunits.*

*Additionally, a heatmap of the entire channel with all resolved positions is attached in the supplementary data to provide an overview. The numbering follows the uniport canonical sequence CAC1D_HUMAN. The less structured N-terminal at the start of the map is unfortunately not well resolved, one may a need sequence ensemble with more conserved N-terminals to detail its impact. Transmembrane domains annotated according to uniport (entry CAC1D_Human, accessed on 03012024) are generally predicted to have higher functional impacts. Inside each domain the S4 helices, pore loop near the selectivity filter and the lower part of the pore region, close to the activation gate have the highest density of predicted pathogenic mutations. In contrast, the majority of the interdomain linkers mutations have little impact. However, residues locate near the beginning of voltage sensor or at the end of the pore domain may organize into additional gating regulators or interact with other proteins. A prime example of such construct is a conserved proline rich domain (corresponding to residue E831 to residue L851 in the heatmap) binding to the A-Kinase anchoring protein (AKAPs)*^39,40^*, which locates near the end of the DII-DIII linker. The proximal end of the C-terminal domain is rather conserved, containing EF hand PreIQ and IQ domain*^41,42^*. The PCRD motif*^42,43^ *from L1640 to E1666 remains visible, however, resolution decreases from this point towards more distal C-terminal. (In summary, we provide a pathogenicity map with residue resolution for most of the known functional sites. Thanks to the good alignment within the L-type calcium channel family, the transmembrane domains are especially well resolved. However, more aligned sequences are needed for the distal terminal regions.)*


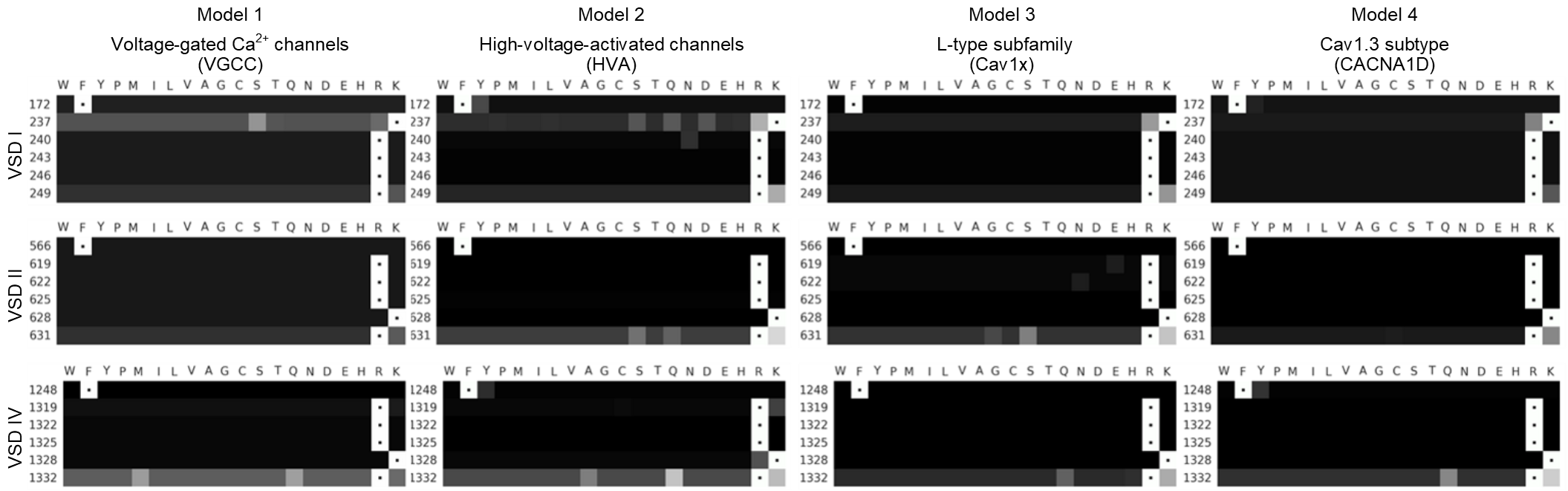


***Supplemental Figure 5: S4 charges and phenylalanine in other domains.***

*Heatmap of S2 phenylalanine residues (Phe; F) forming the voltage-sensors' hydrophobic constriction sites and the positively charged gating residues within the S4 helices in voltage sensors I, II and IV. Phe is always completely conserved in all cases shown. The outermost (first) and innermost (last) positive gating charges within the extracellular/intracellular water-filled cavities of the voltage-sensors can tolerate more variations.*

## **Benchmark on other datasets**

*
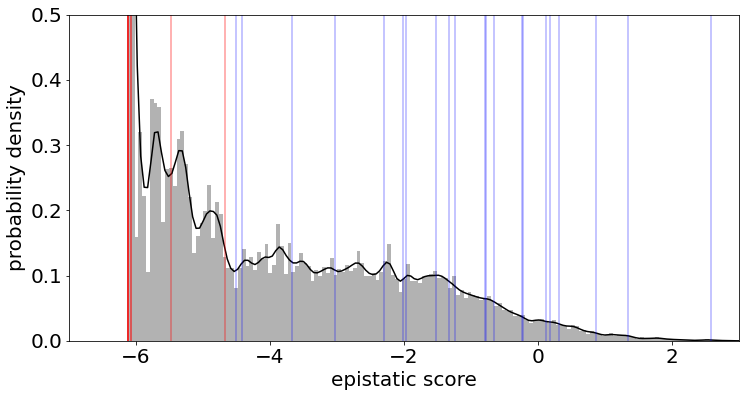

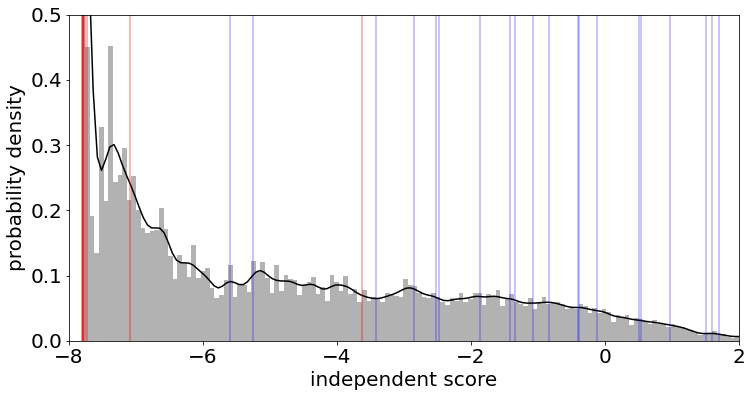
*


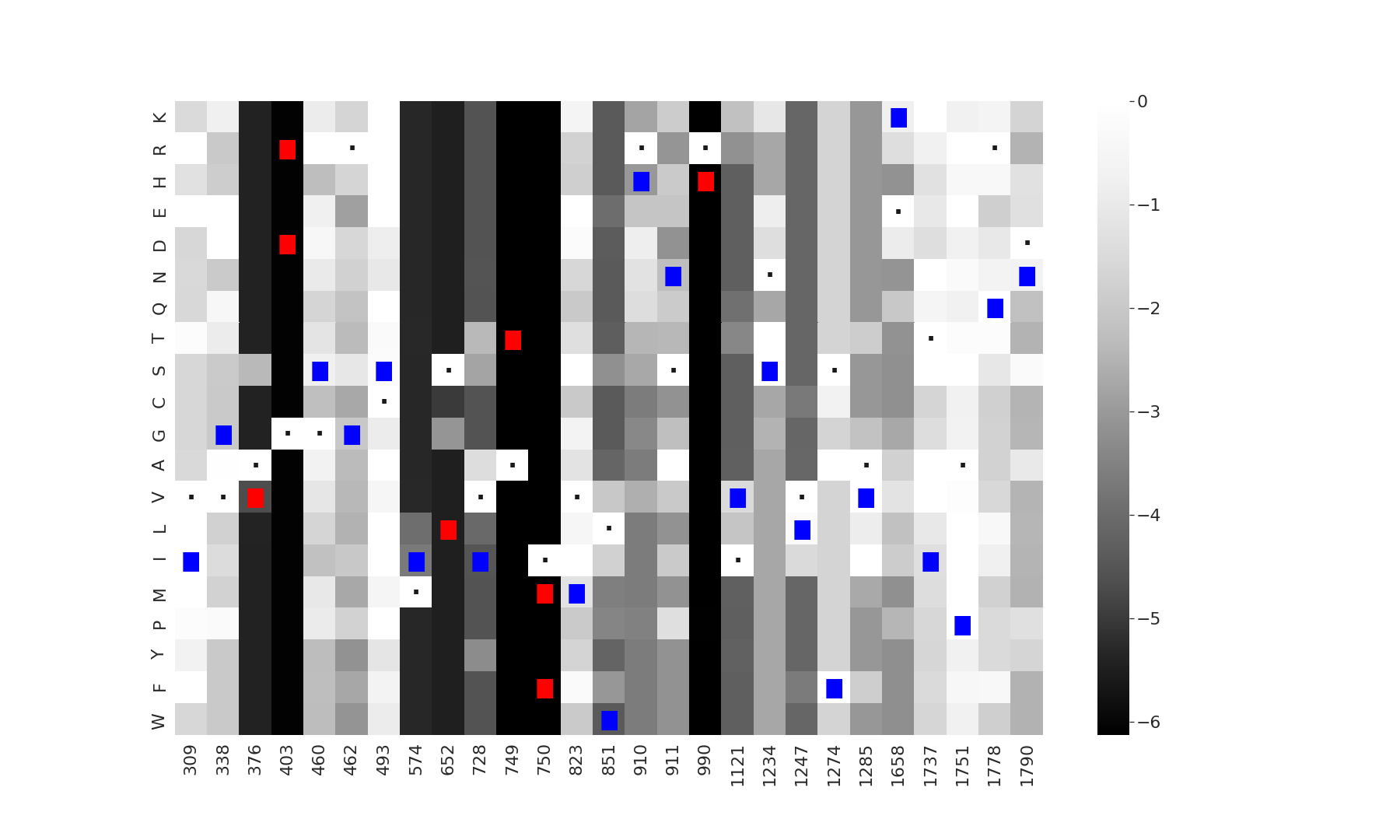
***Supplemental Figure 6: Predictions for the ClinVar Miner dataset by the L-type model (model 3).*** *The upper histograms display performances of the epistatic (left) and independent models (right) on clinVar Miner of general Cav1.3 channelopathies. Variants were classified into (likely) pathogenic (red) and (likely) benign (blue). These variants are mapped into histograms of the scores of all possible variants calculated for the Cav1.3 a1-subunit sequence as described in Methods (aa 68-1888; shown in grey) to correlate the predicted pathogenicity with epistatic scores. The lower heatmap of epistatic scores shows consequences of amino acid changes to different residues at the position of ClinVar Miner variants. Each column denotes one position while rows represent different amino acid residues at the corresponding position. Resulting boxes are colored according to the epistatic score of the sequence. The darkness reflects the statistical energy for the mutant to appear, i.e., severity of the predicted impact. As an example, a dark gray box represents a rather unlikely mutant, i.e., predicted with highly negative impact. A lighter box represents a more evolutionarily favorable mutation, i.e., with less negative epistatic score. Boxes corresponding to the reference wild type sequence are always white and noted with dots. Recorded ClinVar Miner variants are noted in squares with colors corresponding to annotations on pathogenicity, i.e., (likely) pathogenic (red) and (likely) benign (blue).*

*
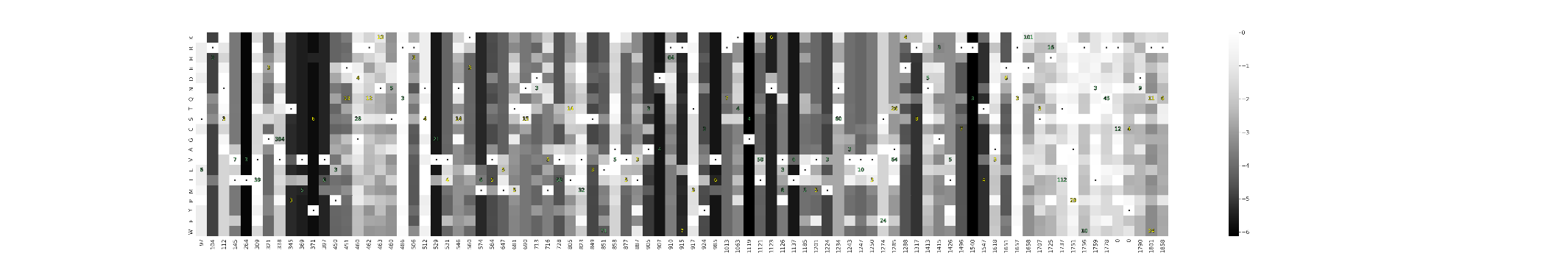
*

***Supplemental Figure 7: heatmap of mutations collected in gnomAD (allele count>3).***

*The figure shows allele counts of repetitively detected gnomAD*^44^ *mutations (allele count>3) mapped into the heatmap of epistatic scores of all possible mutations at the affected sites. Each box represents a mutation at a detected site, with color of the box denotes the epistatic score. Like in Fig.4, a dark gray/black box represents a rather unlikely mutant, i.e., predicted with highly negative impact. A green box represents an evolutionarily favorable mutation, i.e., with positive epistatic score. Boxes corresponding to the reference wild type sequence are always white and noted with dots. The gnomAD mutations are marked with their allele counts (green or orange if annotated as “variant of uncertain significance” in the ClinVar*^45^ *database). Residues numbered 0 locate on the alternative exon 44, not expressed in isoform EU363339, but in both the Uniprot canonical isoform Q01668 and the gnomAD canonical isoform NM_000720. Respectively they hold mutations R1825C and Y1826C according to the NM_000720 numbering.*

Despite limitations caused by clinically silent heterozygous loss of functions mutations, we tested if the epistatic score correlates with the allele count in the gnomAD database assuming that variants reported more frequently in control populations should be more likely to have a less negative epistatic score. Moreover, we identified several variants that appeared more than 2 times, but their negative epistatic scores together with their position in the sequence strongly point to their pathogenic potential (SI. Tab.1)

Variants that appeared more than 2 times with epistatic scores more negative than -4.6 are collected in Table 2 with further details from gnomAD. Although pathogenicity cannot be directly confirmed, for two variants further evidence for a potential pathogenicity could be obtained: V985I is located within the IIIS4 voltage-sensor helix and adjacent gating charges are pathogenic in Cav1.1 and Cav2.1^46^. In addition, R1317 is a conserved positive charge at the cytoplasmic end of the IVS4 voltage-sensor helix and the corresponding gating charge, when mutated to a proline (R1673P) was reported in Cav2.1 α1 in a patient with global developmental delay and cerebellar atrophy^47,46^.

All variants found in large number of healthy controls (allele count>10) are attributed neutral scores, i.e., predicted to be benign. (V529G, or V549 with gnomAD residue number has been removed shortly after our calculation, as it failed the gnomAD quality control.) Predicted pathogenic variants with an allele count > 2 often score near the benign zone, with exceptions of G1119S (allele count 4, MAF 3.66E-5) and R1540Q (allele count 3, MAF 2.74E-5) predicted to be pathogenic. In total, 17 of the 149 mutations with allele count > 2 in the gnomAD control group fall into the more pathogenic zone of our model (<-4.6). All 17 mutations possess low allele frequency (~1E-5, probability to be found in tested controls). Moreover, most of them are marked as of “uncertain significance” by ClinVar. In the SI file gnomAD_score_-4.5_count1.xlsx also all variants below an epistatic score of -4.6 and an allele count of ≤ are given.

| **Protein (gnomAD)** | **Protein (EU363339)** | **ClinVar Clinical Significance** | **Allele Count** | **Allele Frequency** | **Homozygote Count** | **Fraction**  **(sequence aligned)** | **independent score** | **epistatic score** |
| --- | --- | --- | --- | --- | --- | --- | --- | --- |
| R104H | R104H |  | 3 | 2.74E-05 | 0 | 0.72 | -6.49 | -4.89 |
| I264V | I264V |  | 3 | 2.74E-05 | 0 | 1.00 | -7.11 | -5.98 |
| T345P | T345P | Uncertain significance | 3 | 2.49E-05 | 0 | 0.83 | -6.93 | -5.27 |
| V369M | V369M |  | 5 | 4.57E-05 | 0 | 0.81 | -5.66 | -5.38 |
| Y371S | Y371S | Uncertain significance | 6 | 5.48E-05 | 0 | 0.98 | -7.45 | -5.88 |
| V549G | V529G |  | 21 | 1.93E-04 | 0 | 0.85 | -7.15 | -5.63 |
| V604I | V584I | Uncertain significance | 5 | 4.57E-05 | 0 | 0.70 | -5.89 | -4.65 |
| S869L | S849L | Uncertain significance | 3 | 2.74E-05 | 0 | 0.77 | -5.80 | -4.68 |
| D927A | D907A |  | 4 | 3.66E-05 | 0 | 0.95 | -7.35 | -5.71 |
| R935W | R915W | Uncertain significance | 7 | 6.40E-05 | 0 | 0.82 | -5.99 | -5.29 |
| Y944C | Y924C |  | 3 | 2.74E-05 | 0 | 0.70 | -6.42 | -4.74, near IIS2 countercharge |
| V1005I | V985I | Uncertain significance | 6 | 5.48E-05 | 0 | 0.92 | -4.89 | -5.34; IIIS4; V1347M in Cav2.1/DEE |
| G1139S | G1119S |  | 4 | 3.66E-05 | 0 | 1.00 | -7.73 | -6.12 |
| N1143K | N1123K | Uncertain significance | 6 | 5.48E-05 | 0 | 0.85 | -7.15 | -5.47 |
| R1352S | R1317S | Uncertain significance | 8 | 7.31E-05 | 0 | 0.78 | -6.73 | -5.13 S4 Arg in L-types |
| R1575Q | R1540Q |  | 3 | 2.74E-05 | 0 | 1.00 | -7.65 | -6.11 |
| T1582I | T1547I | Uncertain significance | 4 | 3.66E-05 | 0 | 0.74 | -6.58 | -5.22 |

***Supplemental Table 1: Predicted likely pathogenic gnomAD variants with allele count >2.***

*The table presents predicted pathogenic (epistatic score < -4.6) mutations collected from gnomAD controls (allele count > 2), healthy at the moment of test and conditions of their carriers. For an easier comparison, we map the gnomAD mutations to the EU363339 variant, the reference in Ortner et al. study. Allele count and frequency records time and frequency of occurrence in tested controls. Homozygous attendees are missing for all mutations listed, as the count goes to 0. Complimentarily, ClinVar adds notes from the clinical side. The fraction of sequence aligned at tested positions (>= 0.70) are mostly promising for reliable predictions and the independent model agrees with the epistatic model for severe cases (for epistatic score < -5.8 cases, independent score < -7.*

## **Reassessments of 80 previously reported APAs/APCCs mutations**

|  | Steady-state activation | | | | Steady-state inactivation | | | | Inactivation Kinetics: Remaining I_Ca_ [%] | | | | | | |
| --- | --- | --- | --- | --- | --- | --- | --- | --- | --- | --- | --- | --- | --- | --- | --- |
|  | V_0.5_  (mV) | Slope (mV) | V_rev_  (mV) | n | V_0.5_  (mV) | Slope (mV) | Non-inactivating  (%) | n | r_50_ | r_100_ | r_250_ | r_500_ | r_1000_ | r_5000_ | n |
| WT_L_ | 0.59 ± 1.05 | 9.44 ± 0.17 | 67.19 ± 0.98 | 33 | -17.50 ± 0.90 | 5.86 ± 0.19 | 18.52 ±  1.41 | 26 | 66.32 ± 3.00 | 54.32 ± 3.13 | 36.38 ± 2.75 | 24.73 ± 2.23 | 16.94 ± 1.73 | 7.87 ± 0.97 | 21 |
| V584I_L_ | -3.07 ± 1.25* | 8.72 ± 0.11** | 63.55 ± 1.54* | 17 | -18.17 ± 0.88 | 5.29 ± 0.22 | 17.87 ±  1.57 | 15 | 66.11 ± 3.17 | 54.62 ± 3.15 | 38.06 ± 2.75 | 26.36 ± 2.28 | 19.03 ± 1.92 | 9.94 ± 1.44 | 15 |

***Supplemental Table 2: Parameters for voltage-dependent steady-state activation and inactivation as well as inactivation kinetics of the V584I CACNA1D variant.***

*Steady-state activation/inactivation: Parameters were obtained from fitting normalized activation curves (G/G_max_) or normalized steady-state inactivation curves (I/I_control_) to a Boltzman relationship as described previously*^31^*. Inactivation Kinetics: r-values represent the fraction of remaining I_Ca_ after 50, 100, 250, 500, 1,000 and 5,000 ms during a 5-s depolarization to the voltage of maximal inward current (V_max_). All values are presented as mean ± SEM and originate from >3 independent transfections. Statistics: unpaired Student ́s t-test: V584I_L_ in comparison to WT_L_. n, number of experiments. V_0.5_, half maximal activation/inactivation voltage; V_rev_, reversal potential; WT, wild-type.*

| **variant NM_000720** | **variant EU363339** | **ES** | **Variant in other VGCC isoform** | **Pathogenic *CACNA1D* variant(s) in same adrenal** | **Additional evidence** | **Further evidence for pathogenicity (P) or non-pathogenicity (NP) by AC, yes/no (Y/N)** |
| --- | --- | --- | --- | --- | --- | --- |
| **Likely pathogenic** | | | | | | |
| V123A | V123A | -4.99 |  |  |  | - |
| E124K | E124K | -5.37 |  | V1338M^48^ (-6.11) |  | P-N |
| **R240C** | **R240C** | **-6.06** | **R195K in Cav2.1 α1 causing FHM1**^49^ |  |  | **P-Y** |
| L272R | L272R | -5.40 |  | V401L (-5.26), F747L (-6.12), F747V (-6.11), R990H (-6.12), F1248L^48^ (-6.13)) |  | P-N |
| V309A | V309A | -1.54 |  |  | V309 tolerates a large number of replacements (Some even enhance evolutionary fitness based on ES; Fig. 4) (e.g., V309I 84 entries in gnomAD) | NP-Y (likely benign) ^#^ |
| G323R | G323R | -5.32 |  | L248F^48^ (-6.06) |  | P-N |
| E412D | E412D | -6.10 |  | G403R (-6.08), S652L (-5.48), F747V (-6.11), R993S (-6.14), A998V^50^ (-6.13) |  | P-N |
| P568L | P548L | -5.00 |  |  |  | - |
| M590I | M570I | -6.12 |  |  |  | - |
| L633Q | L613Q | -5.34 |  | F747L^51^ (-6.12) |  | P-N |
| **R639P** | **R619P** | **-5.94** | **R583Q in Cav2.1 α1 causing FHM1, ataxia (Kraus et al.**^52^**; Bürk et al.**^53^**)** |  |  | **P-Y** |
| **I670N** | **I650N** | **-6.14** | **I603L in Cav2.3 α1 causing EIEE69 (Helbig et al.**^54^**)** |  |  | **P-Y** |
| L673P | L653P | -6.14 |  | G403R (-6.08), F747V (-6.11), S969L (-5.74), F1248L^48^ (-6.13) |  | P-N |
| S744L | S724L | 0.01 |  | V1338M^48^ (-6.11) | S724 tolerates a large number of replacements (Some even enhance evolutionary fitness based on ES; Fig. 4) | NP-Y (likely benign) ^#^ |
| Y761C | Y741C | -5.51 |  |  |  | - |
| **L768S** | **L748S** | **-6.13** |  | **G403R (-6.08), S652L (-5.48), F747L (-6.12), F747V (-6.11), R990H (-6.12), F1147L**^48^ **(-6.12)** | **L768P causes pathogenic gating change in Cav1.3 (Tadross et al.**^55^**)** | **P-Y** |
| **V772G** | **V752G** | **-6.11** | **V714A in Cav2.1 α1 causing FHM1**^52^ | **G403R (-6.08), F747L (-6.12), R990H**^50^ **(-6.12)** |  | **P-Y** |
| **N774S** | **N754S** | **-6.06** |  |  | **N774P causes pathogenic gating change in Cav1.3**^55^ **(Tadross et al.)** | **P-Y** |
| V999D | V979D | -5.39 |  |  |  | - |
| **K1001N** | **K981N** | **-5.78** | **K1343Q in Cav2.1 α1 causing FHM1**^56^ |  |  | **P-Y** |
| **C1027R** | **C1007R** | **-6.13** | **C1041R in Cav1.2 α1 causing Timothy Syndrome (Nugud et al.**^57^**; C1369R in Cav2.1 α1 causing FHM1 (Thomsen et al.**^58^**)** |  |  | **P-Y** |
| **A1031T** | **A1011T** | **-6.12** | **S1373L in Cav2.1 α1 causing epileptic encephalopathy (Byers et al.**^59^**)** | **F747V**^48^ **(-6.11)** |  | **P-Y** |
| **V1168M** | **V1148M** | **-6.13** | **V1182L in Cav1.2 α1 causing ASD**^60^**; V1427M in Cav3.3 causing ASD**^60^ | **M1354I**^61^ **(-6.12)** |  | **P-Y** |
| **I1172N** | **I1152N** | **-6.13** | **I1186V/T in Cav1.2 α1 cause LQT8 (Wemhöner er al.**^62^**)** |  |  | **P-Y** |
| R1203H | R1183H | -4.10 |  | L248F^48^ (-6.06) |  | P-N |
| D1293N | D1273N | -6.13 |  | S652L (-5.48), R990H (-6.12), I1015V (-5.91), V1338M^48^ (-6.11) |  | P-N |
| **I1387T** | **I1352T** | **-5.51** | **I1709T in Cav2.1 α1 causing FHM1**^63^ |  |  | **P-Y** |
| T1411M | T1376M | -5.60 |  |  |  | - |
| V1482L | V1447L | -6.10 |  |  |  | - |
| P1534L | P1499L | -6.13 |  |  |  | - |
| G1562R | G1527R | -6.13 |  |  |  | - |
| T1879I | T1835I | -0.37 |  | G403R^48^ (-6.08) | T1835 tolerates a large number of replacements (Some even enhance evolutionary fitness based on ES; Fig. 4) | NP-Y (likely benign) ^#^ |
| **likely benign or uncertain significance** | | | | | | |
| G457R | G457R | -1.56 |  | F1248L^64^ (-6.13) | G457 tolerates a large number of replacements (One even enhances evolutionary fitness based on ES; Fig. 4); 1x in gnomAD | NP-Y (likely benign) ^#^ |
| **V604I** | **V584I** | **-4.65** | **evidence for small pathogenic gating change (this paper)** |  | **Small pathogenic gating changes (Fig. 3)** | **P-Y (risk enhancing) *** |
| **R639W** | **R619W** | **-5.94** | **R583Q in Cav2.1 α1 causing FHM1, ataxia (Kraus et al.**^52^**; Bürk et al.**^53^**)** | **F747L**^51^ **(-6.12)** |  | **P-Y** |
| V748I | V728I | -4.51 |  |  |  | - |

***Supplemental Table 3: Re-examination of CACNA1D variants previously classified as "likely pathogenic" or of "uncertain significance".***

*Since the negative epistatic score (ES) suggests pathogenic potential for most of these variants, we screened the literature for indirect evidence that these variants could indeed be pathogenic (AC additional clinical evidence). Such evidence was obtained either from mutations reported in homologous positions of the identical residue in the α1-subunit of another VGCC isoform, pathogenic gating changes reported in site-directed mutations of Cav1.3 α1-subunits or from the presence of pathogenic CACNA1D variants reported in APA/APCC samples of the same adrenal. Findings were summarized in the second-last column as either further evidence supporting (yes, Y) or not supporting (no, N) pathogenicity (P) or non-pathogenicity (NP). Variants for which further evidence supporting a pathogenic role were found are highlighted in bold. For variants identified in APAs/APCCs from the same adrenal, only variants that were classified as clearly pathogenic were included. Residue numbers are given for the CACNA1D genome reference sequence used in the gnomAD database (NM_000720) and in our previous publications (EU363339), which differ due to the incorporation of different exons. Residue numbers for other VGCC isoforms are according to genome reference sequences used previously*^46^*.* ^#^ *Positive epistatic scores for the described variant as well as most other substitutions at this residue (Fig. 4) together with further evidence supporting non-pathogenicity, NP (Y), suggest a likely benign nature. * For V584I we could show typical pathogenic gating changes (activation at more negative voltages; Fig. 3), however, to a minor extent compared to clearly pathogenic variants. We therefore rather propose a risk-enhancing effect with low penetrance for this variant. With arbitrarily chosen cutoffs, mutations scored below -5.8 are considered very pathogenic by the epistatic model, above -3.5 are considered rather benign, scores in between are risk enhancing.*

## **6. Benchmark with existing methods (supplementary data: benchmark with existing methods)**

**Comparison to the polyphen-2 (**<http://genetics.bwh.harvard.edu/pph2/index.shtml>**, accessed on 30.12.2023 and 19.12.2023)**

Our model outperforms PolyPhen-2^65^ in distinguishing between (likely) pathogenic and (likely) benign variants, as shown in the supplementary data benchmark with existing methods (Table 1). In the ClinVar Miner^66^ dataset (https://clinvarminer.genetics.utah.edu/, accessed on 29.11.2022), our model clearly separates the different classes in SI Fig. 6, Polyphen-2 predicts three of the (likely) benign variants (rs201471889, rs186968009, rs115066564) to be pathogenic. All three variants are documented as (likely) benign in UniProtKB^67^ and appear frequently in large population sets (section ‘Frequency’), as documented in the reference SNP (rs) report of the dbSNP^68^ (https://www.ncbi.nlm.nih.gov/snp/, accessed on 02.01.2024) and ClinVar Miner (accessed on 02.01.2024). The rs201471889 variant has a frequency of 2868/115874 in ExAC. The rs186968009 variant not only has high frequencies in dbSNP-documented databases but was also reported by six submitters in ClinVar Miner, all of whom marked it as (likely) benign. The rs115066564 variant has a frequency of over 1E-4 in all exome-sequencing databases documented in dbSNP. A detailed benign report is available at the clinVar Miner. It is important to note that the performance of polyphen-2 remains strong in our benchmark, as well as in other types of benchmarks. It may even be superior to most predictors available, particularly in predicting new variants without functional assays or allele frequency data in humans. For instance, our model for the APAs set provides predictions that are very similar to those of the standalone PolyPhen-2. However, PolyPhen-2 slightly overpredicts the pathogenicity of the risk-enhancing variant V584I (see supplementary data: benchmark with existing methods-table 2).

**Comparison to other popular methods listed as major tools in VEP**

Among the common methods listed in the Ensembl database's Variant Effect Predictor^69^ (VEP) (<https://www.ensembl.org/Homo_sapiens/Tools/VEP>, accessed dates are provided in the table), our method outperforms all but the VEP-specific version of PolyPhen-2^10,69^ (see supplementary data: benchmark with existing methods-table 3, which also include performance of the CADD^70^ and SIFT^71^ integrated in VEP^69^). However, most of the variants that are difficult to predict appear in the training set of PolyPhen-2 integrated in VEP. The VEP testing variants comprise two main parts: variants from the previously collected nucleotide changes in the APAs set, and three difficult-to-predict (likely) benign variants from the benchmark using existing methods, Table 1. The effects of all variants are assessed using the transcript 'ENST00000350061.11', which corresponds to the UniProt canonical sequence CAC1D_HUMAN carrying exon 8B. Of the APA variants, we highlight the V584I in bold as one of the mildest pathogenic cases due to its low disease penetrance in the population. The pathogenicity of G457R and V728I is also weak, although not as well characterized as V584I for a reference point in more detailed comparison among predictions. Most of the tested methods predicted that the pathogenicity level of V584I was similar to or even higher than that of confirmed pathogenic mutations (highlighted in yellow). Only EVE^15^ and PolyPhen^10,69^ (VEP PolyPhen-2) predicted it to be less pathogenic than other confirmed pathogenic APAs variants. Although the two methods scored most of the tested variants reasonably, the relative pathogenicity and coverage of the whole set with clear decisions are not optimal. EVE predicts the class of most variants correctly, however, a considerable amount of confirmed pathogenic variants are marked uncertain or not covered by the prediction. Among them scoring of A998V, and I1015V even fall within the low pathogenicity range. In addition, it may have overlooked the weak pathogenicity of V584I and ranked it as completely benign. In contrast, VEP PolyPhen-2 is more closely aligned with updated APAs annotations and correlates well with ClinVar Miner annotations. It only overpredicts the pathogenicity of one tested variant, L851W.

In contrast to the standalone Polyphe-2, which is based on physics and comparative considerations (<http://genetics.bwh.harvard.edu/pph2/index.shtml>, accesses on 06.01.2024), the VEP Polyphen-2 was trained on UniProtKB variants with annotations that integrate allele frequency data from large screening databases^67,69^. Especially, the uniprotKB already includes the three (likely) benign variants wrongly predicted as pathogenic by the polyphen-2 standalone version independent of the VEP. Given that the uniprotKB built up training set of the polyphen-2 in VEP (<http://genetics.bwh.harvard.edu/pph2/dokuwiki/overview#prediction> accessed 05.01.2024), the good performance of the VEP polyphen-2 predictions may not be transferable to new variants. Although the VEP PolyPhen-2 is helpful in integrating UniProtKB information for commonly known variants, we expect our model to be more effective in predicting the pathogenicity of new variants.

Additionally, many methods employ multiple descriptors with weights that are fine-tuned by specific training sets using machine learning. As a result, the contribution of each considered factor is ambiguous for the final prediction. Our method solely utilizes evolutionary information, providing a complementary perspective to existing methods. It can also be combined with biophysics and clinical annotations to further enhance accuracy. Using merely evolutionary information, we completely avoid data leakage leading to train-test contamination, i.e., self-fulfilling prophecies due to prediction of variants that already were in the training set.

## **7. References:**

1 Draelos RL, Ezekian JE, Zhuang F *et al.* GENESIS: Gene-Specific Machine Learning Models for Variants of Uncertain Significance Found in Catecholaminergic Polymorphic Ventricular Tachycardia and Long QT Syndrome-Associated Genes. *Circ: Arrhythmia and Electrophysiology* 2022; **15**. doi:10.1161/CIRCEP.121.010326.

2 Boßelmann CM, Hedrich UBS, Lerche H, Pfeifer N. Predicting functional effects of ion channel variants using new phenotypic machine learning methods. *PLoS Comput Biol* 2023; **19**: e1010959.

3 Zhang H, Xu MS, Fan X, Chung WK, Shen Y. Predicting functional effect of missense variants using graph attention neural networks. *Nat Mach Intell* 2022; **4**: 1017–1028.

4 Boßelmann CM, Hedrich UBS, Müller P *et al.* Predicting the functional effects of voltage-gated potassium channel missense variants with multi-task learning. *eBioMedicine* 2022; **81**: 104115.

5 Brunklaus A. Advances in genotype-phenotype associations for CACNA1A-related epilepsies. *European Journal of Paediatric Neurology* 2021; **33**: A2.

6 Brünger T, Pérez-Palma E, Montanucci L *et al.* Conserved patterns across ion channels correlate with variant pathogenicity and clinical phenotypes. *Brain* 2023; **146**: 923–934.

7 Peterson TA, Doughty E, Kann MG. Towards Precision Medicine: Advances in Computational Approaches for the Analysis of Human Variants. *Journal of Molecular Biology* 2013; **425**: 4047–4063.

8 Niroula A, Vihinen M. Variation Interpretation Predictors: Principles, Types, Performance, and Choice. *Human Mutation* 2016; **37**: 579–597.

9 Hopf TA, Ingraham JB, Poelwijk FJ *et al.* Mutation effects predicted from sequence co-variation. *Nat Biotechnol* 2017; **35**: 128–135.

10 Adzhubei I, Jordan DM, Sunyaev SR. Predicting Functional Effect of Human Missense Mutations Using PolyPhen‐2. *CP Human Genetics* 2013; **76**. doi:10.1002/0471142905.hg0720s76.

11 Sim N-L, Kumar P, Hu J, Henikoff S, Schneider G, Ng PC. SIFT web server: predicting effects of amino acid substitutions on proteins. *Nucleic Acids Research* 2012; **40**: W452–W457.

12 Hecht M, Bromberg Y, Rost B. Better prediction of functional effects for sequence variants. *BMC Genomics* 2015; **16 Suppl 8**: S1.

13 Hopf TA, Ingraham JB, Poelwijk FJ *et al.* Mutation effects predicted from sequence co-variation. *Nat Biotechnol* 2017; **35**: 128–135.

14 Zhang Y, Grimwood AL, Hancox JC, Harmer SC, Dempsey CE. Evolutionary coupling analysis guides identification of mistrafficking-sensitive variants in cardiac K+ channels: Validation with hERG. *Front Pharmacol* 2022; **13**: 1010119.

15 Riesselman AJ, Ingraham JB, Marks DS. Deep generative models of genetic variation capture the effects of mutations. *Nat Methods* 2018; **15**: 816–822.

16 Wiuf A, Steffen JH, Becares ER *et al.* The two-domain elevator-type mechanism of zinc-transporting ZIP proteins. *Sci Adv* 2022; **8**: eabn4331.

17 Kotler E, Shani O, Goldfeld G *et al.* A Systematic p53 Mutation Library Links Differential Functional Impact to Cancer Mutation Pattern and Evolutionary Conservation. *Mol Cell* 2018; **71**: 178-190.e8.

18 Akyuz N, Karavitaki KD, Pan B *et al.* Mechanical gating of the auditory transduction channel TMC1 involves the fourth and sixth transmembrane helices. *Sci Adv* 2022; **8**: eabo1126.

19 Gofman Y, Schärfe C, Marks DS, Haliloglu T, Ben-Tal N. Structure, Dynamics and Implied Gating Mechanism of a Human Cyclic Nucleotide-Gated Channel. *PLoS Comput Biol* 2014; **10**: e1003976.

20 Nicoludis JM, Gaudet R. Applications of sequence coevolution in membrane protein biochemistry. *Biochimica et Biophysica Acta (BBA) - Biomembranes* 2018; **1860**: 895–908.

21 Palovcak E, Delemotte L, Klein ML, Carnevale V. Comparative sequence analysis suggests a conserved gating mechanism for TRP channels. *J Gen Physiol* 2015; **146**: 37–50.

22 Toombes GES, Swartz KJ. Divining the design principles of voltage sensors. *J Gen Physiol* 2014; **143**: 139–144.

23 Kasimova MA, Lynagh T, Sheikh ZP *et al.* Evolutionarily Conserved Interactions within the Pore Domain of Acid-Sensing Ion Channels. *Biophys J* 2020; **118**: 861–872.

24 Palovcak E, Delemotte L, Klein ML, Carnevale V. Evolutionary imprint of activation: the design principles of VSDs. *J Gen Physiol* 2014; **143**: 145–156.

25 Granata D, Ponzoni L, Micheletti C, Carnevale V. Patterns of coevolving amino acids unveil structural and dynamical domains. *Proc Natl Acad Sci U S A* 2017; **114**: E10612–E10621.

26 Elbahnsi A, Delemotte L. Structure and Sequence-based Computational Approaches to Allosteric Signal Transduction: Application to Electromechanical Coupling in Voltage-gated Ion Channels. *J Mol Biol* 2021; **433**: 167095.

27 Cabezas-Bratesco D, Mcgee FA, Colenso CK *et al.* Sequence and structural conservation reveal fingerprint residues in TRP channels. *Elife* 2022; **11**: e73645.

28 Wu J, Yan Z, Li Z *et al.* Structure of the voltage-gated calcium channel Cav1.1 at 3.6 Å resolution. *Nature* 2016; **537**: 191–196.

29 Ortner NJ, Kaserer T, Copeland JN, Striessnig J. De novo CACAN1D Ca2+ channelopathies: clinical phenotypes and molecular mechanism. *Pflugers Arch - Eur J Physiol* 2020; **472**: 755–773.

30 Pinggera A, Negro G, Tuluc P, Brown MJ, Lieb A, Striessnig J. Gating defects of disease-causing de novo mutations in Cav1.3 Ca2+ channels. *Channels (Austin)* 2018; **12**: 388–402.

31 Török F, Tezcan K, Filippini L *et al.* Germline de novo variant F747S extends the phenotypic spectrum of CACNA1D Ca2+ channelopathies. *Hum Mol Genet* 2023; **32**: 847–859.

32 Yao X, Gao S, Yan N. Structural basis for pore blockade of human voltage-gated calcium channel Cav1.3 by motion sickness drug cinnarizine. *Cell Res* 2022; **32**: 946–948.

33 Costé de Bagneaux P, Campiglio M, Benedetti B, Tuluc P, Flucher BE. Role of putative voltage-sensor countercharge D4 in regulating gating properties of CaV1.2 and CaV1.3 calcium channels. *Channels (Austin)* 2018; **12**: 249–261.

34 Fernández-Quintero ML, El Ghaleb Y, Tuluc P, Campiglio M, Liedl KR, Flucher BE. Structural determinants of voltage-gating properties in calcium channels. *eLife* 2021; **10**: e64087.

35 Ortner NJ. CACNA1D-Related Channelopathies: From Hypertension to Autism. *Handb Exp Pharmacol* 2023. doi:10.1007/164_2022_626.

36 Catterall WA. Voltage-gated calcium channels. *Cold Spring Harb Perspect Biol* 2011; **3**: a003947.

37 Fernández-Quintero ML, El Ghaleb Y, Tuluc P, Campiglio M, Liedl KR, Flucher BE. Structural determinants of voltage-gating properties in calcium channels. *Elife* 2021; **10**: e64087.

38 Gao S, Yao X, Yan N. Structure of human Cav2.2 channel blocked by the painkiller ziconotide. *Nature* 2021; **596**: 143–147.

39 Altier C, Dubel SJ, Barrere C *et al.* AKAP79 modulation of L-type channels involves disruption of intramolecular interactions in the CaV1.2 subunit. *Channels (Austin)* 2012; **6**: 157–165.

40 Altier C, Dubel SJ, Barrère C *et al.* Trafficking of L-type calcium channels mediated by the postsynaptic scaffolding protein AKAP79. *J Biol Chem* 2002; **277**: 33598–33603.

41 Striessnig J, Pinggera A, Kaur G, Bock G, Tuluc P. L-type Ca2+ channels in heart and brain. *Wiley Interdiscip Rev Membr Transp Signal* 2014; **3**: 15–38.

42 Singh A, Gebhart M, Fritsch R *et al.* Modulation of voltage- and Ca2+-dependent gating of CaV1.3 L-type calcium channels by alternative splicing of a C-terminal regulatory domain. *J Biol Chem* 2008; **283**: 20733–20744.

43 Tan BZ, Jiang F, Tan MY *et al.* Functional characterization of alternative splicing in the C terminus of L-type CaV1.3 channels. *J Biol Chem* 2011; **286**: 42725–42735.

44 Chen S, Francioli LC, Goodrich JK *et al.* A genome-wide mutational constraint map quantified from variation in 76,156 human genomes. Genetics, 2022 doi:10.1101/2022.03.20.485034.

45 Landrum MJ, Lee JM, Benson M *et al.* ClinVar: improving access to variant interpretations and supporting evidence. *Nucleic Acids Res* 2018; **46**: D1062–D1067.

46 Striessnig J. Voltage-Gated Ca2+-Channel α1-Subunit de novo Missense Mutations: Gain or Loss of Function – Implications for Potential Therapies. *Front Synaptic Neurosci* 2021; **13**: 634760.

47 Tyagi S, Bendrick TR, Filipova D, Papadopoulos S, Bannister RA. A mutation in CaV2.1 linked to a severe neurodevelopmental disorder impairs channel gating. *Journal of General Physiology* 2019; **151**: 850–859.

48 Omata K, Satoh F, Morimoto R *et al.* Cellular and Genetic Causes of Idiopathic Hyperaldosteronism. *Hypertension* 2018; **72**: 874–880.

49 Ducros A, Denier C, Joutel A *et al.* The clinical spectrum of familial hemiplegic migraine associated with mutations in a neuronal calcium channel. *N Engl J Med* 2001; **345**: 17–24.

50 Yamazaki Y, Nakamura Y, Omata K *et al.* Histopathological Classification of Cross-Sectional Image-Negative Hyperaldosteronism. *J Clin Endocrinol Metab* 2017; **102**: 1182–1192.

51 Nishimoto K, Tomlins SA, Kuick R *et al.* Aldosterone-stimulating somatic gene mutations are common in normal adrenal glands. *Proc Natl Acad Sci U S A* 2015; **112**: E4591-4599.

52 Kraus RL, Sinnegger MJ, Koschak A *et al.* Three New Familial Hemiplegic Migraine Mutants Affect P/Q-type Ca2+ Channel Kinetics. *Journal of Biological Chemistry* 2000; **275**: 9239–9243.

53 Bürk K, Kaiser FJ, Tennstedt S *et al.* A novel missense mutation in CACNA1A evaluated by in silico protein modeling is associated with non-episodic spinocerebellar ataxia with slow progression. *Eur J Med Genet* 2014; **57**: 207–211.

54 Helbig KL, Lauerer RJ, Bahr JC *et al.* De Novo Pathogenic Variants in CACNA1E Cause Developmental and Epileptic Encephalopathy with Contractures, Macrocephaly, and Dyskinesias. *Am J Hum Genet* 2018; **103**: 666–678.

55 Tadross MR, Johny MB, Yue DT. Molecular endpoints of Ca2+/calmodulin- and voltage-dependent inactivation of Cav1.3 channels. *Journal of General Physiology* 2010; **135**: 197–215.

56 Stam A, Vanmolkot K, Kremer H *et al.* CACNA1A R1347Q: a frequent recurrent mutation in hemiplegic migraine. *Clinical Genetics* 2008; **74**: 481–485.

57 Nugud AA, ELkholy NM, Omar AA *et al.* Case Report: Expanding the Phenotypic Spectrum of Timothy Syndrome Type 1: A Sporadic Case With a de novo CACNA1C Pathogenic Variant and Segmental Ileal Dilatation. *Front Pediatr* 2021; **9**: 634655.

58 Thomsen LL, Kirchmann M, Bjornsson A *et al.* The genetic spectrum of a population-based sample of familial hemiplegic migraine. *Brain* 2007; **130**: 346–356.

59 Byers HM, Beatty CW, Hahn SH, Gospe SM. Dramatic Response After Lamotrigine in a Patient With Epileptic Encephalopathy and a De NovoCACNA1A Variant. *Pediatr Neurol* 2016; **60**: 79–82.

60 Zhou X, Feliciano P, Shu C *et al.* Integrating de novo and inherited variants in 42,607 autism cases identifies mutations in new moderate-risk genes. *Nat Genet* 2022; **54**: 1305–1319.

61 Nishimoto K, Koga M, Seki T *et al.* Immunohistochemistry of aldosterone synthase leads the way to the pathogenesis of primary aldosteronism. *Mol Cell Endocrinol* 2017; **441**: 124–133.

62 Wemhöner K, Friedrich C, Stallmeyer B *et al.* Gain-of-function mutations in the calcium channel CACNA1C (Cav1.2) cause non-syndromic long-QT but not Timothy syndrome. *J Mol Cell Cardiol* 2015; **80**: 186–195.

63 Beauvais K, Cavé-Riant F, De Barace C, Tardieu M, Tournier-Lasserve E, Furby A. New CACNA1A Gene Mutation in a Case of Familial Hemiplegic Migraine with Status epilepticus. *Eur Neurol* 2004; **52**: 58–61.

64 Omata K, Anand SK, Hovelson DH *et al.* Aldosterone-Producing Cell Clusters Frequently Harbor Somatic Mutations and Accumulate With Age in Normal Adrenals. *J Endocr Soc* 2017; **1**: 787–799.

65 Adzhubei IA, Schmidt S, Peshkin L *et al.* A method and server for predicting damaging missense mutations. *Nat Methods* 2010; **7**: 248–249.

66 Henrie A, Hemphill SE, Ruiz-Schultz N *et al.* ClinVar Miner: Demonstrating utility of a Web-based tool for viewing and filtering ClinVar data. *Hum Mutat* 2018; **39**: 1051–1060.

67 The UniProt Consortium, Bateman A, Martin M-J *et al.* UniProt: the Universal Protein Knowledgebase in 2023. *Nucleic Acids Research* 2023; **51**: D523–D531.

68 Sherry ST, Ward M, Sirotkin K. dbSNP-database for single nucleotide polymorphisms and other classes of minor genetic variation. *Genome Res* 1999; **9**: 677–679.

69 McLaren W, Gil L, Hunt SE *et al.* The Ensembl Variant Effect Predictor. *Genome Biol* 2016; **17**: 122.

70 Kircher M, Witten DM, Jain P, O’Roak BJ, Cooper GM, Shendure J. A general framework for estimating the relative pathogenicity of human genetic variants. *Nat Genet* 2014; **46**: 310–315.

71 Ng PC, Henikoff S. Predicting deleterious amino acid substitutions. *Genome Res* 2001; **11**: 863–874.

Figure 1 contains animal representation pictures adapted from "Zebrafish" by DataBase Center for Life Science (DBCLS) (https://doi.org/10.7875/togopic.2021.004), used under CC BY 4.0, "Amphioxus" by Giovanni Maki (https://doi.org/10.1371/journal.pbio.0030219), used under CC BY-SA 3.0, "Caenorhabditis elegans" by Zeynep F. Altun (https://commons.wikimedia.org/wiki/File:Adult_Caenorhabditis_elegans.jpg), used under CC BY-SA2.5 and "Golfball sponge" by John Turnbull (https://www.flickr.com/photos/johnwturnbull/15336294245/), used under CC BY-SASA 2.0 and other pictures from the common domain.
